# Subunit-specific analysis of cohesin-mutant myeloid malignancies reveals distinct ontogeny and outcomes

**DOI:** 10.1038/s41375-024-02347-y

**Published:** 2024-07-20

**Authors:** Johann-Christoph Jann, Christopher B. Hergott, Marisa Winkler, Yiwen Liu, Benjamin Braun, Anne Charles, Kevin M. Copson, Shougat Barua, Manja Meggendorfer, Niroshan Nadarajah, Shai Shimony, Eric S. Winer, Martha Wadleigh, Richard M. Stone, Daniel J. DeAngelo, Jacqueline S. Garcia, Torsten Haferlach, R. Coleman Lindsley, Marlise R. Luskin, Maximilian Stahl, Zuzana Tothova

**Affiliations:** 1 Department of Medical Oncology, Dana-Farber Cancer Institute, Boston, MA 02215 USA; 2 Cancer Program, Broad Institute, Cambridge, MA 02142 USA; 3 Department of Pathology, Brigham and Women’s Hospital, Boston, MA 02115 USA; 4 Element Iowa City (JMI Laboratories), North Liberty, IA 52317 USA; 5 Department of Data Science, Dana-Farber Cancer Institute, Boston, MA 02215 USA; 6 MLL Munich Leukemia Laboratory, Max-Lebsche-Platz 31, 81377 Munich, Germany

**Keywords:** Myelodysplastic syndrome, Epidemiology, Acute myeloid leukaemia

## Abstract

Mutations in the cohesin complex components (*STAG2, RAD21, SMC1A, SMC3*, and *PDS5B)* are recurrent genetic drivers in myelodysplastic neoplasm (MDS) and acute myeloid leukemia (AML). Whether the different cohesin subunit mutations share clinical characteristics and prognostic significance is not known. We analyzed 790 cohesin-mutant patients from the Dana-Farber Cancer Institute (DFCI) and the Munich Leukemia Laboratory (MLL), 390 of which had available outcome data, and identified subunit-specific clinical, prognostic, and genetic characteristics suggestive of distinct ontogenies. We found that *STAG2* mutations are acquired at MDS stage and are associated with secondary AML, adverse prognosis, and co-occurrence of secondary AML-type mutations. In contrast, mutations in *RAD21, SMC1A* and *SMC3* share features with de novo AML with better prognosis, and co-occurrence with de novo AML-type lesions. The findings show the heterogeneous nature of cohesin complex mutations, and inform clinical and prognostic classification, as well as distinct biology of the cohesin complex.

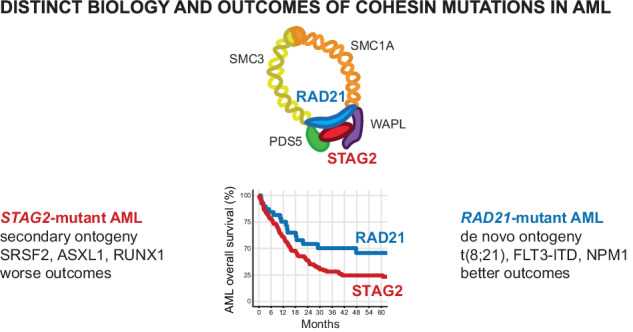

## Introduction

Myeloid neoplasms are a heterogeneous group of clonal disorders of mutant hematopoietic stem and progenitor cells, characterized by diverse clinical presentations and outcomes [1, 2]. Mutations in the family of genes encoding the multimeric protein complex cohesin [3] are present across the spectrum of myeloid neoplasms, including myelodysplastic neoplasm (MDS), myeloproliferative neoplasms (MPN), MDS/MPN overlap syndromes, acute myeloid leukemia (AML), and chronic myeloid leukemia (CML) [4–9]. In human somatic cells, proteins encoded by *SMC1*, *SMC3, RAD21*, and one of the paralog genes *STAG1* or *STAG2*, form a ring-like structure, which wraps around the DNA, and is loaded and unloaded by the modulator proteins MAU2-NIPBL and PDS5-WAPL, respectively [3]. The cohesin complex dynamically shapes genome architecture and regulates gene expression and DNA integrity, and loss of function mutations in the hematopoietic system have shown to effect hematopoietic stem cell self-renewal and differentiation, leading to the development of myeloid neoplasia [10–19].

Cohesin mutations have been understood as genetic drivers in myeloid malignancies for almost a decade, however, detailed examination of the cohesin gene-specific disease characteristics and the prognostic impact are lacking. *STAG2* mutations have previously been grouped with other secondary AML ontogeny-defining mutations [20, 21], and the 2022 European Leukemia Network (ELN) guidelines classify them in the subgroup of adverse risk AML [22]. However, it is unclear whether a secondary AML ontogeny attribution and adverse prognostic impact can also be assigned to the less frequent cohesin complex mutations in *SMC1A*, *SMC3, RAD21*, and *PDS5B* [21]. Furthermore, an independent prognostic value for any cohesin gene mutation has previously not been established [23, 24].

In the largest cohort of cohesin-mutated myeloid neoplasms reported to date, we characterized the incidence, clinical presentation, genomic landscape, and clinical outcomes of cohesin subunit mutations, and identified subunit-specific effects with disease ontogeny and prognostic implications, which informs distinct biology of these important genetic drivers.

## Methods

### Patient cohort

We analyzed 2 independent cohorts of patients as described below (Supplementary Fig. 1). Cohort 1 (“DFCI cohort”) included 5,191 patients seen at the Dana-Farber Cancer Institute (DFCI) with a confirmed hematologic malignancy as defined by the 2016 World Health Organization (WHO) classification [25] from August 2014 to November 2021 based on morphology and cytogenetic findings. For these cases, the WHO diagnoses were retrospectively translated to WHO 2022 [2] classification based on published diagnostic criteria. Classification of all cohesin-mutant (MT) cases underwent independent hematopathology review. The subset of 759 patients with any detectable variant (defined by previously established allele frequency thresholds [26]) in a cohesin complex gene regardless of disease entity were extracted, and 311 (40.1%) patients were found to have a pathogenic cohesin mutation (as defined in the “Mutational profiling” section below). From these, 256 cohesin-MT AML, MDS, or MDS/MPN patients were compared to 3,109 wild type (WT) cases. Patients were compared in terms of demographics, clinical characteristics, and outcomes.

Cohort 2 (“MLL cohort”) included a total of 479 patients treated across Germany who underwent diagnostic workup of a suspected or confirmed myeloid malignancy at the Munich Leukemia Laboratory (MLL) between 2005 and 2022 and were found to have 1 or more pathogenic mutations in *STAG2, SMC1A, SMC3*, or *RAD21* (*PDS5B* mutation status was not assessed). Diagnoses from peripheral blood and bone marrow were made based on cytomorphology, cytogenetics, and molecular genetics as previously described [27–29] in accordance with the 2016 WHO classification and reviewed by 2 board-certified hematopathologists. All cases were classified into specific subgroups according to the WHO 2022 classification. All 479 cases were used for the demographics and disease type analyses. Only 134/479 MLL cases (28%) had sufficient clinical annotation with available date of diagnosis and follow-up for outcome analysis. In addition, a selected cohort of 1378 cases (838 MDS and 540 AML) without evidence of a cohesin mutation and with available follow-up data were selected from the MLL dataset based on comprehensive sequencing data availability and used to compare outcomes. All patients gave their consent for genetic analyses and the use of laboratory results for research purposes. The study was approved by the DFCI Institutional Review Board (IRB) and the MLL IRB.

### Mutational profiling

For the DFCI cohort, cytogenetic data were extracted from clinical reports of karyotype and fluorescent in-situ hybridization (FISH) generated by the DFCI clinical cytogenetics laboratory. Molecular data were obtained from reports of clinical next-generation sequencing (NGS) performed using the DFCI Rapid Heme Panel (RHP) at diagnosis and relapse, as previously reported [26]. Genes included in the RHP were selected based on their known or suspected involvement in the pathogenesis of myeloid or lymphoid cancers, or inherited or acquired bone marrow failure syndromes, and are listed in the Supplementary Table 1. One of two different versions of RHP was used for sequencing analysis of study cases: samples acquired between August 2014-October 2019 were analyzed using RHP version 2, which was based on a custom amplicon-based approach. A minimum of 10 variant reads or 5–9 variant reads at >33% allelic frequency were required for mutation calling. RHP version 3 was used for samples acquired after November 2019, and the revised platform used unique molecular identifiers (UMIs) for error-suppression to allow reliable variant calls at a variant allelic fraction (VAF) of 0.01 or greater requiring a minimum of 3 mutant reads. Median mutation coverage was 568x (95% confidence 100x-2036x). All truncation, frameshift, or splice site mutations in *STAG2, RAD21, SMC1A, SMC3*, and *PDS5B* were considered pathogenic, and all missense mutations were manually reviewed for non-germline allelic frequency and damaging PolyPhen score ( > 0.85), or for evidence in OncoKB [30] or COSMIC [31] for significant mutations in cancer [21]. Mutational and cytogenetic analyses for the MLL cohort were performed as previously reported and based on whole genome sequencing (WGS), and validation used targeted deep sequencing [32, 33]. A total of 763 samples were assayed by whole genome sequencing (WGS) and analyzed as described in previous reports [7, 34, 35]. There were 1157 cases assayed by targeted sequencing, which were analyzed during routine diagnostic workup or for research purposes [7]. WGS data confirmed all mutations detected by targeted NGS panels and was further consulted for completing the mutational analysis of the 73 genes.

### Transcriptomic analysis of BeatAML

Raw counts from patients with reported mutations [36] in *STAG2*, *RAD21*, *SMC3* or *SMC1A* were normalized using DESeq2. Differentially expressed genes were called between any cohesin subunits using DESeq2 with an FDR < 5%. All these genes were used for unsupervised k-means clustering and annotated cohesin mutations were superimposed to this clustering. Genes from each k-means cluster were queried against Metascape [37], and gene sets with FDR < 0.01 were plotted.

### Statistical analysis

All statistical analyses were conducted using R v4.2.1. Statistical significance was considered using a significance level α of 0.05. Normality was assessed using the Shapiro-Wilk test. If normality distribution was met, a two-sided unpaired t-test was applied to continuous variables between 2 groups unless stated otherwise. Non-normal distributed continuous variables between 2 groups were analyzed using a Wilcoxon rank sum test. For multiple group comparison, an ANOVA analysis was first performed, followed by an emmeans test for the indicated comparisons using rstatix package v0.7.1. Mutational co-occurrence was calculated as pairwise odds ratio (OR) for any given gene between patients with the respective cohesin-MT and WT cases. Statistical significance was derived from Fisher exact test, which was adjusted for multiple testing using Benjamini-Hochberg procedure. Outcome analyses were carried out using the Kaplan Meier method stratified by the presence or absence of a respective mutation. Statistical comparison was conducted using Cox proportional hazard ratio (HR) (coxph, survival package v3.2-11) and a two-sided log rank test with default parameters using survfit. For multivariate modeling, only significant univariate parameters were used forward in a Cox proportional model, using transplantation as a time-dependent variable. Unless stated otherwise, only results that hold significance in the merged datasets were reported in the main text; individual cohorts are presented in the supplement.

## Results

### Cohesin-mutant hematologic malignancies have distinct disease characteristics

**Fig. 1:**
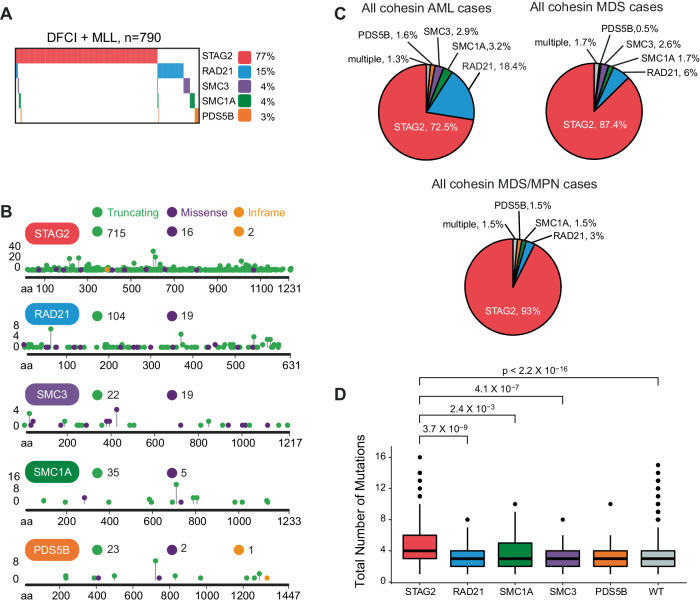
Molecular characterization of cohesin mutations in hematologic malignancies demonstrates subunit-specific differences. **A** Oncoprint of all patients with cohesin mutations (DFCI and MLL cohorts combined), *n* = 790. **B** Lollipop plot panel of cohesin mutations for the combined cohort. **C** Pie charts of distribution of cohesin mutations across MDS, AML, and MDS/MPN for the combined cohort. **D** Box plot of the total number of pathogenic mutations identified by targeted sequencing of patients with cohesin-mutant MDS and AML at the time of diagnosis, stratified by cohesin status for the combined cohort. Wilcoxon test was used to determine significance.

We investigated 2 large cohorts of patients diagnosed with a hematologic malignancy for presence of a mutation in the cohesin complex (Supplementary Fig. 1). In total, we identified 790 patients with a pathogenic mutation in any of the cohesin complex genes (Fig. 1A). The incidence of cohesin mutation was 10% in MDS, 5% in MDS/MPN, and 8% in AML patients in the DFCI cohort (Supplementary Table 2). Mutations in different cohesin subunits were noted to be mutually exclusive with each other, with only 12/790 (2%) cases characterized by mutations in more than 1 cohesin subunit, and they were spread throughout the coding sequence without any hotspots (Fig. 1B and Supplementary Fig. 2). *STAG2* mutations were the most common and present in 610 (77%) of patients, followed by *RAD21* in 104 cases (13%), *SMC3* in 26 cases (3%), *SMC1A* in 22 cases (3%), and *PDS5B* in 16 cases (2%). Frameshift indel mutations were the most frequent type of mutations for most cohesin genes except for *STAG2*, where nonsense mutations leading to a premature stop codon comprised more than 50% of all mutations (Supplementary Fig. 3A). Patients with mutations in the cohesin complex were diagnosed with AML, MDS, and MDS/MPN in 374, 351, and 63 cases, respectively (Table 1, Supplementary Fig. 3B). In the DFCI cohort, we identified 55 patients (17.7%) with a pathologist-validated non-myeloid hematologic malignancy, including indolent and high-grade lymphomas (chronic lymphocytic leukemia (*n* = 7), diffuse large B cell lymphoma (n = 7), multiple myeloma (*n* = 3), and acute lymphoblastic leukemia (*n* = 8)) (Supplementary Fig. 3C). In all subsequent analyses, we excluded cases with non-myeloid hematologic malignancies and focused on patients with AML, MDS, and MDS/MPN only.

**Table 1:** Patient and disease characteristics of cohesin-mutant versus cohesin-wild type MDS, MDS/MPN, and AML patients.

		Cohesin mutant	Wild type	*p* value
	*N*	735	4487	
Sex *n/N (%)*	3860			0.03
Female		237 / 696 (34%)	1197/3,109 (39%)	
Male		459 / 696 (66%)	1912/3,109 (61%)	
Missing Data		39	1378	
Age at… *Median (95% CI)*				
MDS diagnosis	1654	73 (70, 73)	69 (67, 68)	<0.001
AML diagnosis	2621	69 (65, 68)	65 (62, 63)	<0.001
MDS/MPN diagnosis	505	68 (67, 72)	71 (69, 70)	0.55
Cohort *n/N (%)*	5277			<0.001
DFCI		256 / 735 (35%)	3109 / 4487 (69%)	
MLL		479 / 735 (65%)	1378 / 4487 (31%)	
MDS WHO2022, n / N (%)	2495	351 / 735 (48%)	2144 / 4487 (48%)	
MDS-5q		4 / 351 (1.1%)	93 / 2144 (4.3%)	0.004
MDS-biTP53 ^a^		0 / 351 (0%)	70 / 2144 (3.3%)	<0.001
MDS-IB1		124 / 351 (35%)	346 / 2144 (16%)	<0.001
MDS-IB2		125 / 351 (36%)	357 / 2144 (17%)	<0.001
MDS-LB		80 / 351 (23%)	889 / 2144 (41%)	<0.001
MDS-SF3B1		18 / 351 (5.1%)	389 / 2144 (18%)	<0.001
AML WHO2022 *n/N (%)*	2621	374 / 735 (51%)	2247 / 4487 (50%)	
Acute myeloid leukemia with BCR-ABL1		0 / 374 (0%)	5 / 2247 (0.2%)	>0.99
AML by differentiation		31 / 374 (8.3%)	688 / 2247 (31%)	<0.001
AML with biallelic CEBPA		6 / 374 (1.6%)	53 / 2247 (2.4%)	0.36
AML with CBFB-MYH11		2 / 374 (0.5%)	89 / 2247 (4.0%)	<0.001
AML with DEK-NUP214		0 / 374 (0%)	15 / 2247 (0.7%)	0.15
AML with GATA2, MECOM		1 / 374 (0.3%)	64 / 2247 (2.8%)	0.003
AML with KMT2A-MLLT3		1 / 374 (0.3%)	42 / 2247 (1.9%)	0.024
AML with NPM1		43 / 374 (11%)	322 / 2247 (14%)	0.14
AML with PML-RARA		1 / 374 (0.3%)	121 / 2247 (5.4%)	<0.001
AML with RUNX1-RUNX1T1		15 / 374 (4.0%)	54 / 2247 (2.4%)	0.072
AML-MR		274 / 374 (73%)	775 / 2247 (34%)	<0.001
Myeloid Sarcoma		0 / 374 (0%)	19 / 2247 (0.8%)	0.10
Progression ^b^ *n/N (%)*				
cases w/ MDS > AML progression		45/140 (32%)	277 /1304 (21%)	0.005
cases w/o MDS > AML progression		95 /140 (68%)	1027/1304 (68%)	
MDS/MPN *n/N (%)*	505	63 / 735 (9%)	442 / 3649 (12%)	
aCML/BCR-ABL1 negative		10/63 (16%)	30/442 (6.8%)	0.02
CMML		29/63 (46%)	289/442 (65%)	0.005
MDS/MPN-u		21/63 (33%)	64/442 (14%)	<0.001
MDS/MPN with RS-T		0/63 (0%)	44/442 (10.0%)	0.02
Other		3/63 (4.8%)	15/442 (3.4%)	0.8

^a^Defined as VAF > 0.7 or presence of two pathogenic mutations.

^b^DFCI cohort only.

Patients were divided into a cohesin-WT (*n* = 4487) or cohesin-MT (*n* = 735) cohort. We then systematically compared the clinical and demographic features of these 2 cohorts. Cohesin-MT patients were older at the time of diagnosis (AML: 69 vs. 65 years; MDS: 73 vs. 69 years; *p* < 0.0001) and had different patterns of AML and MDS subtypes than their cohesin-WT counterparts. AML with myelodysplasia-related defining genetic abnormalities (AML-MR) was present in 73% of cohesin-MT compared to 34% of cohesin-WT cases (*p* < 0.001). Conversely, AML without genetically defined lesions (summarized as AML by differentiation) and AML with *NPM1* mutation were more common among cohesin-WT than cohesin-MT cases (31% vs. 8.3%, *p* < 0.001; 14 vs. 11%, *p* = 0.14, respectively). Within MDS, the cohesin-MT cohort had a higher fraction of more advanced MDS diagnoses than the cohesin-WT cohort (MDS-IB1: 35% vs. 16%, *p* < 0.0001; MDS-IB2: 36% vs. 17%, *p* < 0.0001). Consistent with these findings, the fraction of patients with documented progression from MDS to AML was higher in MDS patients with cohesin mutations than MDS patients without these mutations (32% vs. 21%, *p* = 0.005; data only available for the DFCI cohort). Notably, MDS with bi-allelic *TP53* inactivation, del5q, and *SF3B1* associated MDS were nearly mutually exclusive with cohesin-mutant MDS (Table 1, Supplementary Table 2). These data demonstrate that cohesin mutations segregate with distinct clinical features linked to MDS and subsequent secondary AML.

### Cohesin complex mutations have distinct clinical features and AML ontogeny

We next aimed to delineate differences among mutations of the cohesin complex components in patients with AML, MDS, and MDS/MPN overlap syndromes (Table 2, Supplementary Table 3). Given their high prevalence, our analysis focused on the comparison between cases with *STAG2* versus *RAD21* mutations, and examined the impact of *SMC1A*, *SMC3*, and *PDS5B* mutations as a group due to their significantly lower numbers (thereafter referred to as *SMC1A/SMC3/PDS5B*). We observed that patients with *STAG2* mutations were older than patients with mutations in other cohesin genes at time of AML diagnosis (70 vs. 64 vs. 64/57/50 years, *p* < 0.001, Supplementary Fig. 3D) but not at time of MDS diagnosis (Supplementary Fig. 3E). We observed a significant difference in the distribution of cohesin subunit mutations, with 72.5% of all AML cases, but 87.4% of all MDS cases (*p* < 0.001) and 93% of MDS/MPN cases (*p* = 0.001) carrying a *STAG2* mutation (Fig. 1C, Table 2). Furthermore, there was a significant difference in the AML ontogeny among different cohesin mutations. *STAG2* mutations were almost exclusively associated with an AML-MR diagnosis (256/271, 94%), with rare cases of *NPM1* and bi-allelic *CEBPA* (8/271 (3%) and 5/271 (1.8%), respectively) (Table 2). Conversely, patients with *RAD21* and *SMC1A/SMC3/PDS5B* mutations presented more frequently with de novo AML with *NPM1* mutations (27/69 (39%), 2/12 (17%), 3/11(27%), and 2/6 (33%) cases, respectively) versus *STAG2*-mutant patients (*p* < 0.0001). Core binding factor leukemia t(8;21)/RUNX1::RUNX1T1 was found in 10/69 (14%) of *RAD21*-mutant patients and 2/29 (7%) of *SMC1A/SMC3/PDS5B*-mutant patients but only in 1/271 (0.4%) *STAG2-*mutant patient (*p* < 0.0001 and 0.026, Table 2).

**Table 2:** Patient and disease characteristics of different cohesin complex mutations in MDS, MDS/MPN, and AML patients.

		STAG2	RAD21	*p* value *STAG2*	SMC1A	SMC3	PDS5B	*p* value *STAG2 v*	Multiple
	*N*	584	92	*v RAD21*	19	20	9	*SMC1A,SMC3,PDS5B*	11
**Sex** *n/N (%)*	**696**			**0.059**				**0.91**	
Female		179 / 547 (33%)	39 / 91 (43%)		4 / 18 (22%)	9 / 20 (45%)	2 / 9 (22%)		4 / 11 (36%)
Male		368 / 547 (67%)	52 / 91 (57%)		14 / 18 (78%)	11 / 20 (55%)	7 / 9 (78%)		7 / 11 (64%)
Missing		37	1		1	0	0		0
**Age at…**Median (95% CI)									
MDS Diagnosis	**348**	72 (70, 73)	73 (64, 75)	0.5	79 (64, 86)	73 (70, 78)	66 (0, 159)	0.34	80 (66, 91)
AML Diagnosis	**374**	70 (68, 71)	64 (56, 64)	<0.001	64 (44, 69)	57 (47, 64)	50 (26, 80)	<0.001	73 (56, 83)
MDS/MPN Diagnosis	**28**	68 (59, 79)	76 (76, 76)		NA	NA	79		21
**Cohort** n/N (%)	**735**			**0.29**				**0.005**	
DFCI		198 / 584 (34%)	26 / 92 (28%)		10 / 19 (53%)	7 / 20 (35%)	9 / 9 (100%)		6 / 11 (55%)
MLL		386 / 584 (66%)	66 / 92 (72%)		9 / 19 (47%)	13 / 20 (65%)	0 / 9 (0%)		5 / 11 (45%)
**MDS WHO2022**, n / N (%)	**351**	**305 / 584 (52%)**	**23 / 92 (25%)**		**6 / 19 (32%)**	**9 / 20 (45%)**	**2 / 9 (22%)**		**6 / 11 (55%)**
MDS-5q		2 / 305 (0.7%)	2 / 23 (8.7%)	0.026	0 / 6 (0%)	0 / 9 (0%)	0 / 2 (0%)	>0.99	0 / 6 (0%)
MDS-IB1		113 / 305 (37%)	6 / 23 (26%)	0.29	0 / 6 (0%)	2 / 9 (22%)	1 / 2 (50%)	0.10	2 / 6 (33%)
MDS-IB2		115 / 305 (38%)	5 / 23 (22%)	0.13	1 / 6 (17%)	2 / 9 (22%)	0 / 2 (0%)	0.095	2 / 6 (33%)
MDS-LB		63 / 305 (21%)	8 / 23 (35%)	0.11	4 / 6 (67%)	2 / 9 (22%)	1 / 2 (50%)	0.046	2 / 6 (33%)
MDS-SF3B1		12 / 305 (3.9%)	2 / 23 (8.7%)	0.26	1 / 6 (17%)	3 / 9 (33%)	0 / 2 (0%)	0.007	0 / 6 (0%)
**AML WHO2022**, n / N (%)	**374**	**271 / 584 (46%)**	**69 / 92 (75%)**		**12 / 19 (63%)**	**11 / 20 (55%)**	**6 / 9 (67%)**		**5 / 11 (45%)**
AML by differentiation		0 / 271 (0%)	22 / 69 (32%)	<0.001	4 / 12 (33%)	1 / 11 (9.1%)	3 / 6 (50%)	<0.001	1 / 5 (20%)
AML with biallelic CEBPA		5 / 271 (1.8%)	1 / 69 (1.4%)	>0.99	0 / 12 (0%)	0 / 11 (0%)	0 / 6 (0%)	>0.99	0 / 5 (0%)
AML with CBFB-MYH11		0 / 271 (0%)	0 / 69 (0%)	NA	1 / 12 (8.3%)	1 / 11 (9.1%)	0 / 6 (0%)	0.009	0 / 5 (0%)
AML with GATA2, MECOM		1 / 271 (0.4%)	0 / 69 (0%)	>0.99	0 / 12 (0%)	0 / 11 (0%)	0 / 6 (0%)	>0.99	0 / 5 (0%)
AML with KMT2A-MLLT3		0 / 271 (0%)	0 / 69 (0%)	NA	1 / 12 (8.3%)	0 / 11 (0%)	0 / 6 (0%)	0.10	0 / 5 (0%)
AML with NPM1		8 / 271 (3.0%)	27 / 69 (39%)	<0.001	2 / 12 (17%)	3 / 11 (27%)	2 / 6 (33%)	<0.001	1 / 5 (20%)
AML with PML-RARA		0 / 271 (0%)	0 / 69 (0%)	NA	0 / 12 (0%)	1 / 11 (9.1%)	0 / 6 (0%)	0.10	0 / 5 (0%)
AML with RUNX1-RUNX1T1		1 / 271 (0.4%)	10 / 69 (14%)	<0.001	1 / 12 (8.3%)	1 / 11 (9.1%)	0 / 6 (0%)	0.026	2 / 5 (40%)
AML-MR		256 / 271 (94%)	9 / 69 (13%)	<0.001	3 / 12 (25%)	4 / 11 (36%)	1 / 6 (17%)	<0.001	1 / 5 (20%)
**MDS/MPN WHO2022,** n / N (%)	**63**	**58 / 584 (10%)**	**2 / 92 (2%)**		**1 / 19 (5%)**	**0 / 20 (0%)**	**1 / 9 (11%)**		**1 / 11 (9%)**
aCML/BCR-ABL1 negative		10/58 (17%)	0/2 (0%)	1	0/1 (0%)	0/0 (NA%)	0/1 (0%)	1	0/1 (0%)
CMML		27/58 (47%)	1/2 (50%)	1	0/1 (0%)	0/0 (NA%)	1/1 (100%)	1	0/1 (0%)
MDS/MPN-u		19/58 (33%)	1/2 (50%)	1	1/1 (100%)	0/0 (NA%)	0/1 (0%)	1	0/1 (0%)
Others		2/58 (3.4%)	0/2 (0%)	1	0/1 (0%)	0/0 (NA%)	0/1 (0%)	1	1/1 (100%)

Consistently, *RAD21* and *SMC1A*/*SMC3*/*PDS5B* mutations represented a significantly greater proportion of cohesin-mutant AML than MDS as compared to *STAG2* mutations (Fig. 1C). This suggests that *STAG2* mutations tend to be acquired at the MDS stage, and *RAD21* and the other cohesin subunit mutations may be more likely acquired at the AML stage and lead to rapid leukemic transformation rather than a slower increase in blast count over time, as may be expected in MDS. Indeed, patients with *RAD21* and *SMC1A/SMC3/PDS5B* mutations trended towards a higher percentage of blasts in their diagnostic AML bone marrow biopsy compared to patients with *STAG2* mutations (median morphology-defined blast count of 47% for *RAD21* vs. 28% for *STAG2*-mutant AML, *p* = 0.11, Supplementary Fig. 3F, data available for the DFCI cohort only).

To further investigate whether *STAG2* mutations may be preferentially acquired at MDS stage and lead to development of secondary AML, we extracted all patients with available longitudinal mutation data and identified 23 patients diagnosed with *STAG2*-mutant AML with at least one mutational assessment before AML diagnosis. The median time from the first mutational assessment to AML diagnosis was 13 months (range 6–53 months) (Supplementary Fig. 4A, B and 5A). Out of 23 cases, 17 patients (73%) had a *STAG2* mutation that was detected prior to AML diagnosis, of which 13 patients (76%) were diagnosed with MDS, and 4 patients (24%) with MDS/MPN. There were 5/23 patients (22%) that showed the first emergence of a *STAG2* mutation at AML diagnosis, and only 1/23 patients (4%) acquired the *STAG2* mutation upon AML disease relapse. Furthermore, we observed a relatively stable *STAG2* VAF leading up to AML diagnosis (Supplementary Fig. 4C, *p* = 0.85), suggesting that the *STAG2*-mutant clone had already dominated the bone marrow in non-leukemic cells before the AML diagnosis was made. These data collectively suggest that the clinical presentation of patients with mutations in different cohesin subunits is not uniform, with different mutations affecting disease biology and AML ontogeny in distinct ways. Furthermore, our data demonstrate that *STAG2* mutations do not act as AML-defining lesions and are usually acquired at the MDS stage.

### Cohesin complex mutations are associated with unique co-mutational, cytogenetic and transcriptional profiles

**Fig. 2:**
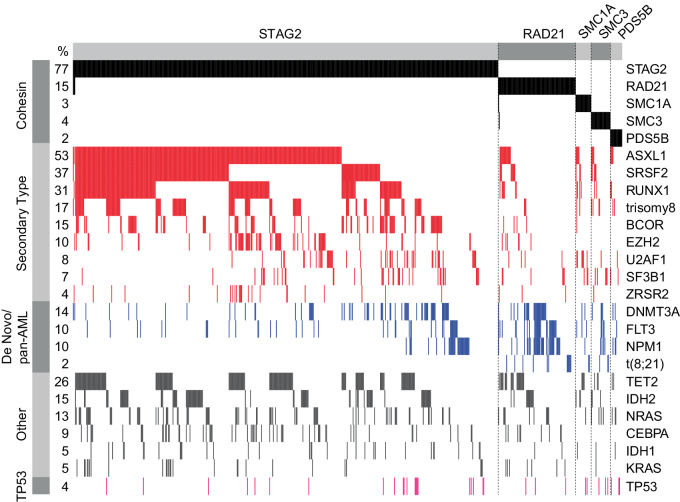
Co-mutational patterns suggest distinct AML ontogeny for *STAG2* versus *RAD21, SMC3, SMC1A* and *PDS5B* mutations. Oncoprint for MDS, AML, and MDS/MPN patients from the combined cohort. Cases with *STAG2*, *RAD21, SMC3, SMC1A*, and *PDS5B* mutations were sorted by co-mutational pattern based on disease ontogeny [21] and association with cohesin subunits. A 2% allelic frequency cut off was used.

**Fig. 3:**
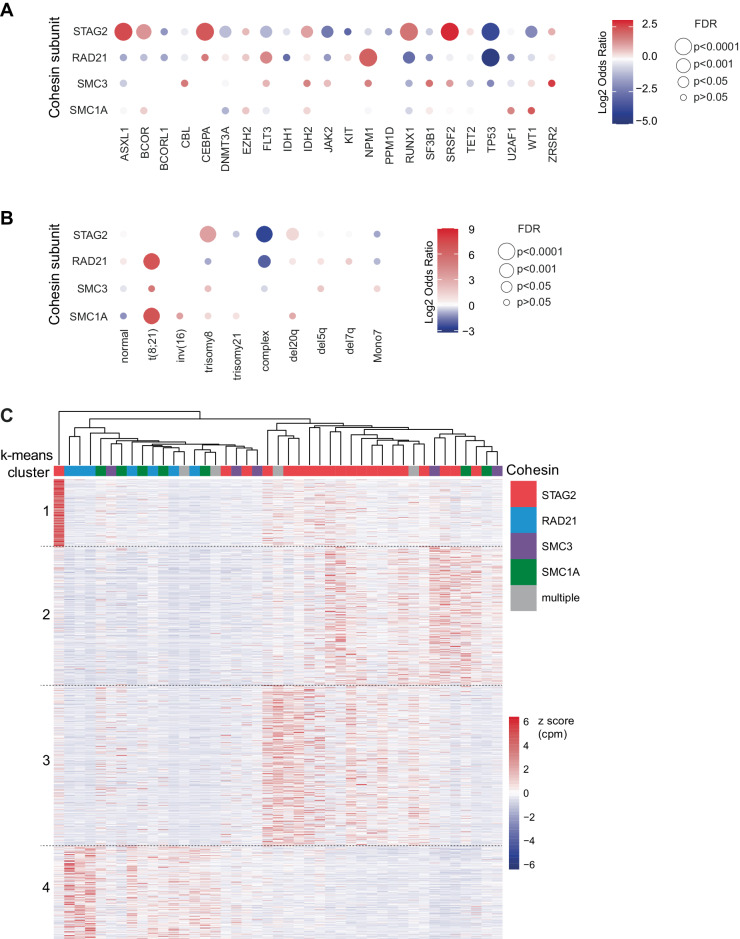
Mutations in different cohesin subunits display unique mutational and chromosomal abnormality characteristics. Balloon plot for relative enrichment of co-occurrence of cohesin subunit mutations with other myeloid driver mutations (**A**) and chromosomal aberrations (**B**). Cohesin-WT cohort was used as reference to calculate enrichment, which is indicated as log2 odds ratio (OR). Combinations with q < 0.05 or 5% mutational frequency in the total cohort are shown in (**A**). Missing balloons indicate OR = 0. False discovery rate (FDR) is as indicated: ***<0.0001, **<0.001, *<0.05 and corresponds to dot size. **C** Gene expression heatmap of cohesin mutant patients with annotated cohesin mutations and clustered by k-means (1328 genes). Samples were clustered by hierarchical clustering and annotated by cohesin mutation. Color indicate z score transformed CPM per gene.

Having established a unique pattern of disease characteristics of cohesin mutations, we next examined the genetic characteristics associated with each mutation. To dissect the genetic makeup of *STAG2* and other cohesin gene mutations, we first compared the total number of additional detected mutations. We observed that patients with *STAG2* mutations had a higher number of co-mutations compared to cohesin-WT patients (median 4 vs. 3, *p* < 0.001), and all other cohesin mutations (median 4 vs. 3, *p* < 0.003, Fig. 1D, Supplementary Fig. 3H). Next, we analyzed differences in the co-mutational patterns between *STAG2* and *RAD21*-mutant AML and MDS patients (Fig. 2, Supplementary Fig. 5–6). The most frequently co-occurring mutations with *STAG2* were secondary type mutations, including mutations in *ASXL1* (64%), *SRSF2* (45%) and *RUNX1* (37%). In contrast, *RAD21* mutations were much more likely to co-occur with de novo or pan-AML mutations, including *NPM1* (32%) and *FLT3* (23%). We also observed t(8;21) in 14% of *RAD21*-mutated cases, with and without additional *KIT* mutations, but not in a single case of *STAG2*-mutant disease. By systematically comparing the co-mutational landscape of *STAG2* mutations and other cohesin gene mutations (Fig. 3A), we found a significant positive enrichment of *STAG2* being co-mutated with secondary ontogeny-defining mutations, including *ASXL1, SRSF2, BCOR*, and *RUNX1* (OR = 5.6, 7.5, 2.5, 3.5; false discovery rate (FDR)= 4×10^–29^, 5×10^–31^, 7×10^–5^, 2×10^–13^, respectively; Supplementary Table 4, Supplementary Fig. 7A-B). Conversely, mutations in *NPM1* and *DNMT3A* were underrepresented in patients carrying a *STAG2* mutation (OR = 0.7 and 0.5; FDR = 0.5 and 0.004, respectively). In contrast, the co-mutational pattern for *RAD21*, *SMC3*, and *SMC1A* was distinct from *STAG2*, and confirmed enrichment for de novo and pan AML ontogeny-defining mutations *NPM1* and *FLT3* (OR = 4.4 and 2.3; FDR = 1×10^–5^ and 0.01, respectively). Interestingly, *STAG2* and *RAD21* mutations shared a near-mutational exclusivity with *TP53* mutations (OR = 0.04–0.06, FDR < 0.001), which was evident for MDS and AML cases (OR_STAG2-MDS_ = 0.07, OR_STAG2-AML_ = 0.05, OR_RAD21-MDS_ = 0, OR_RAD21-AML_ = 0.08).

We similarly observed a distinct pattern of co-occurring cytogenetic aberrations among cohesin-MT cases (Fig. 3B, Supplementary Fig. 7C, D). Trisomy 8 was enriched in *STAG2*-mutant versus cohesin-WT cases (OR = 10.6, FDR = 2.2×10^–50^). In contrast, mutations in *RAD21, SMC1A*, and *SMC3* were enriched for t(8;21) (OR = 134, 131, 37; FDR = 4.1×10^–19^, 3.8×10^–5^, 0.14, respectively). Complex karyotypes were less common among all cohesin-MT patients compared to cohesin-WT patients (OR_STAG2_ = 0.12, FDR = 2.1×10^–22^; OR_RAD21_ = 0.24, FDR = 0.006), which would be expected given their near-mutual exclusivity with *TP53* mutations.

Our genetic and cytogenetic analyses supported the hypothesis that *STAG2-* and non-*STAG2-* mutant myeloid diseases represent distinct biology and ontogeny. To investigate whether this was supported by distinct gene expression programs, we analyzed transcriptomic profiles of the cohesin-mutant AML cases in the BeatAML cohort [36]. Using unsupervised k-means clustering, we observed distinct transcriptional profiles of *STAG2*- and *RAD21/SMC3/SMC1A*-mutant cases (Fig. 3C). Gene set enrichment analysis (GSEA) highlighted differential expression of viral response and interferon signaling, as well as metabolic programs and extracellular matrix-associated pathways (Supplementary Fig. 8). In summary, the distinct co-mutational, cytogenetic and molecular landscapes of different cohesin mutations suggest unique patterns of disease development driven by different cohesin mutations.

### *STAG2* mutations have prognostic impact in MDS and AML

**Fig. 4:**
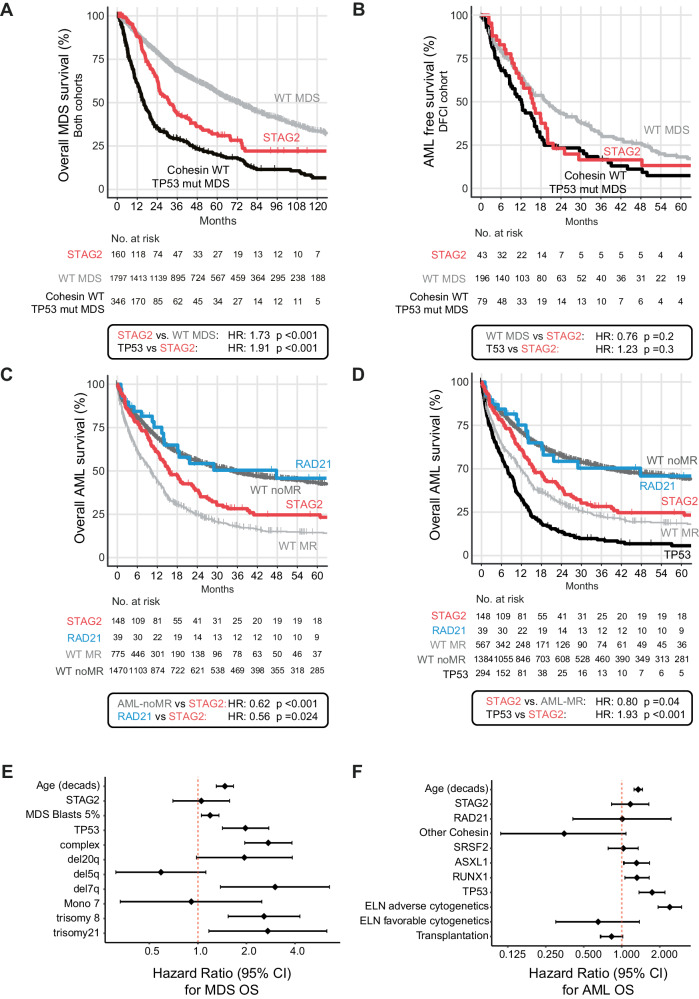
Prognostic impact of *STAG2* mutations in MDS and AML as secondary ontogeny mutations. Survival analysis using the Kaplan-Meier method and log-rank test for overall survival in MDS (**A**), AML-Progression free survival in MDS (DFCI cohort only) (**B**), and AML survival stratified by cohesin subunit mutational status and cohesin-WT group by AML MR or AML-non-MR (**C**) and *TP53* mutation (**D**). HR = Hazard ratio. Statistical significance was determined using the log rank-test. **E** Forrest plot for multivariate prognostic impact of *STAG2* mutations for MDS OS and (**F**) for AML OS.

Having established unique disease and genetic characteristics for different cohesin mutations, we next assessed their impact on clinical outcomes. We conducted independent analyses of overall survival (OS) and progression free survival (PFS) in MDS and AML. The median follow-up time for the entire patient cohort was 73.8 months (95% confidence interval (CI) = 69.6–80.5 months) for MDS, and 49.4 months (95% CI = 45.4–54.4 months) for AML. We first compared outcomes for *STAG2*-mutant MDS to cohesin-WT MDS in which *STAG2* conferred a poor risk at a median OS of 30.3 versus 58.9 months (HR: 1.44, 95% CI 1.17–1.78, *p* < 0.001, combined cohort, Supplementary Fig. 9). Given our observations of near-mutual exclusivity of cohesin and *TP53* mutations (Figs. 2 and 3A), and the well-established association of *TP53* mutations with poor outcomes [38, 39], we next compared the OS and AML-free survival of patients with *STAG2*-mutant MDS to *TP53-*mutant MDS and cohesin/*TP53-*WT MDS. We observed a significantly worse OS of *STAG2*-mutant MDS compared to cohesin/*TP53-*WT MDS (HR = 1.73, 95% CI = 1.4–2.14, median OS 30.3 vs. 69.8 months, *p* < 0.001, Fig. 4A), and a similar risk of leukemic transformation in *STAG2-* and *TP53*-mutant MDS cases (median AML-PFS of 15.4 months for *STAG2* and 12.1 months for *TP53*, *p* = 0.3, DFCI cohort only, Fig. 4B). In a multivariable regression analysis to ascertain the effect of mutations, cytogenetics, diagnostic blast count, and age at the time of MDS diagnosis, the presence of a *STAG2* mutation did not reach significance as an independent predictor of MDS outcome (Fig. 4E).

For our outcome analysis in AML, we first compared *STAG2-*mutant AML to cohesin-WT cases separated into cohesin-WT AML associated with myelodysplasia-related changes (thereafter referred to as “AML-MR”) and cohesin-WT AML not associated with myelodysplasia-related changes (thereafter referred to as “AML-non-MR”) according to the WHO 2022 classification. We observed that *STAG2-*mutant AML had significantly worse OS than AML-non-MR (HR = 0.62, 95% CI = 0.5–0.76, median OS 16 vs. 35 months, *p* < 0.001), and only a modestly better OS than AML-MR (HR = 1.43, 95% CI = 1.16–1.77, median OS 10.3, *p* < 0.001, Fig. 4C). Given the near-mutual exclusivity of *STAG2* and *TP53* mutations in AML, we performed a subset analysis of cohesin-WT AML excluding *TP53*-mutant cases which removed most of the differences and showed a very similar and numerically even favorable outcome between *STAG2*-mutant and AML-MR without *TP53* mutations. (HR = 0.80, 95% CI = 0.64–0.99, median OS 13.6 vs. 11.8 months, *p* = 0.04, Fig. 4D). This poor outcome was also evident for the rare *STAG2*-mutant cases that were not diagnosed as AML-MR because of competing classifying mutations (e.g., *NPM1* and/or *CEPBA*, Supplementary Fig. 10A, B).

Importantly, this pattern was distinct from the outcomes of *RAD21-*mutant AML, which was almost identical to AML-non-MR and significantly better than *STAG2*-mutant AML OS (HR = 0.56, 95% CI = 0.34–0.93, median OS 48 vs. 16 months *p* = 0.024) (Fig. 4C, Supplementary Fig. 11A, B). This effect was most apparent in the DFCI cohort, although we observed the same trend in the MLL cohort, with differences likely being driven by intrinsic variability in treatment and selection biases between DFCI and MLL (Supplementary Fig. 11C, D). Allogenic stem cell transplantation cases accounted for 41% of DFCI but only 12% of MLL cases (Supplementary Table 5), and response rates to induction therapy (Supplementary Table 6) were similar between groups. The effects of *STAG2* and *RAD21* mutations on OS remained significant when censored for allogeneic stem cell transplantation (Supplementary Fig. 10B, Supplementary Fig. 12), although neither one reached statistical significance as an independent predictor of outcome in a multivariable regression analysis of known clinical co-variables (age and transplantation as time dependent variables) and co-mutation with *ASXL1*, *SRSF2*, *RUNX1* and *TP53* (Fig. 4F).

In summary, our findings suggest that only *STAG2* mutations confer a negative impact on AML outcomes, which is attributed to secondary ontogeny and a genetic makeup of preceding myeloid dysplasia. Notably, the prognostic impact of *RAD21* mutations is shared with de novo AML.

## Discussion

Our study establishes a role for different cohesin subunit mutations in distinct subtypes of MDS and AML, which has significant prognostic implications, expands our current understanding of this important group of driver genes, and informs unique biology of different cohesin subunits. We assembled and analyzed the largest existing cohort of 790 patients with cohesin-mutant hematologic malignancies and demonstrated that mutations in *STAG2* and *RAD21* shape the presentation and outcome of AML in unique ways, which can be explained by distinct co-mutational patterns and AML ontogeny. Furthermore, the size of our cohort strongly supports this prognostic impact to be driven by disease ontogeny in both MDS and AML, which was under-appreciated in significantly smaller cohorts [23, 24].

We demonstrated that *STAG2* mutations are associated with secondary AML ontogeny, are usually acquired at MDS or MDS/MPN stage, and co-occur with other secondary ontogeny-defining mutations, such as *ASXL1, SRSF2*, and *RUNX1*. Our data are in agreement with initial reports identifying *STAG2* as one of the eight secondary AML ontogeny defining lesions [21], as well as the 2022 International Consensus Classification which uses *STAG2* as an AML-MR defining mutation for classification of AML [40, 41]. In contrast, we found that *RAD21* mutations are associated with de novo AML, are rarely preceded by MDS or MDS/MPN, and are associated with de novo or pan AML molecular abnormalities, such as t(8;21), *FLT3*, and *NPM1* mutations [24]. These differences are also reflected in distinct gene expression patterns between *STAG2-* and non-*STAG2-*mutant AML, and the unique co-mutational and cytogenetic patterns likely contribute to distinct biological trajectories of leukemic evolution and warrant further investigation in preclinical models.

Importantly, clinical outcomes reflect the different ontogeny associated with *STAG2* and non-*STAG2* cohesin mutations, including *RAD21, SMC1A*, and *SMC3* mutations. While we did not find them to be independent prognostic markers, the distinct pattern of outcomes is reflective of their different disease ontogeny. We observed that *STAG2* mutations conferred overall survival similar to AML-MR, while *RAD21-*mutant cases displayed overall survival similar to AML-non-MR cases. In addition, *SMC1A* and *SMC3-*mutant cases similarly share clinical and molecular features with AML-non-MR. We therefore propose that *RAD21, SMC1A*, and *SMC3-*mutant AML should be considered as AML-non-MR. Our data demonstrate that within the family of cohesin complex mutations, only *STAG2* mutations are indicative of secondary ontogeny and are associated with worse clinical outcomes.

We observed that *STAG2*-mutant cases may have higher numbers of co-mutations (as determined by targeted sequencing panels, with its inherent limitations), which could be a clinical proxy of an intrinsically increased genomic instability. This is in line with several prior studies demonstrating that *STAG2* deficiency is coupled with replication fork stalling, impaired DNA damage repair, and accumulation of DNA damage [14, 42–44]. These findings have contributed to the therapeutic window for inhibitors of Poly(ADP-ribose)polymerase (PARP) [14], which are currently being investigated in a proof of concept study of single agent and combination treatment with hypomethylating agents in a clinical trial for cohesin-mutant AML and MDS (Clinicaltrials.gov identifier NCT03974217). Furthermore, the association of *STAG2-*mutations with trisomy 8 is intriguing considering recent findings suggesting that *RAD21* is the driver of chromosome 8 gain to mitigate replication stress in Ewing sarcoma [45], a disease characterized by frequent *STAG2* mutations. Currently, our data does not allow us to predict the order of *STAG2* and trisomy 8 acquisition, or whether trisomy 8 may affect response to DNA damage repair inhibitors or replication fork stressors, such as PARP inhibitors or hydroxyurea.

The strengths of our study include the large, well-annotated patient cohorts that were representative of clinical practice in Europe and the United States, although such retrospective analyses have an inherent selection bias found in both cohorts to different extents. We therefore note that the retrospective nature of this approach limits the generalizability of our results. Furthermore, the numbers of *SMC1A* and *SMC3-*mutant cases in our cohort were significantly smaller than the number of *STAG2* and *RAD21-*mutant cases, which may limit some of our conclusions about mutations in these cohesin subunits.

In summary, our study contributes to a better understanding of the distinct effects of cohesin gene mutations in myeloid malignancies. Although the biology underlying these differences is not yet known, our work supports the notion that not all cohesin subunit mutations were created equal and that the distinct pattern of cohesin mutations across cancer types may be driven by the unique biology of cohesin subunits.

### Supplementary information

Supplementary Figures

Supplementary Table 1

Supplementary Table 2

Supplementary Table 3

Supplementary Table 4

Supplementary Table 5

Supplementary Table 6

## Data Availability

The datasets generated during and/or analyzed during the current study are available from the corresponding author on request.

## References

[1] Bernard E, Tuechler H, Greenberg PL, Hasserjian RP, Ossa JEA, Nannya Y, et al. Molecular International Prognostic Scoring System for Myelodysplastic Syndromes. NEJM Evid. 2022;1:EVIDoa2200008. 38319256 10.1056/EVIDoa2200008

[2] Khoury JD, Solary E, Abla O, Akkari Y, Alaggio R, Apperley JF, et al. The 5th edition of the World Health organization classification of haematolymphoid tumours: myeloid and histiocytic/dendritic neoplasms. Leukemia. 2022;36:1703–19. 35732831 10.1038/s41375-022-01613-1 PMC9252913

[3] Losada A. Cohesin in cancer: chromosome segregation and beyond. Nat Rev Cancer. 2014;14:389–93. 24854081 10.1038/nrc3743

[4] Kon A, Shih LY, Minamino M, Sanada M, Shiraishi Y, Nagata Y, et al. Recurrent mutations in multiple components of the cohesin complex in myeloid neoplasms. Nat Genet. 2013;45:1232–7. 23955599 10.1038/ng.2731

[5] Cancer Genome Atlas Research N, Ley TJ, Miller C, Ding L, Raphael BJ, Mungall AJ, et al. Genomic and epigenomic landscapes of adult de novo acute myeloid leukemia. N. Engl J Med. 2013;368:2059–74. 23634996 10.1056/NEJMoa1301689 PMC3767041

[6] Jann JC, Tothova Z. Cohesin mutations in myeloid malignancies. Blood. 2021;138:649–61. 34157074 10.1182/blood.2019004259 PMC8394903

[7] Haferlach T, Nagata Y, Grossmann V, Okuno Y, Bacher U, Nagae G, et al. Landscape of genetic lesions in 944 patients with myelodysplastic syndromes. Leukemia. 2014;28:241–7. 24220272 10.1038/leu.2013.336 PMC3918868

[8] Papaemmanuil E, Gerstung M, Malcovati L, Tauro S, Gundem G, Van Loo P, et al. Clinical and biological implications of driver mutations in myelodysplastic syndromes. Blood. 2013;122:3616–27. 24030381 10.1182/blood-2013-08-518886 PMC3837510

[9] Patel BJ, Przychodzen B, Thota S, Radivoyevitch T, Visconte V, Kuzmanovic T, et al. Genomic determinants of chronic myelomonocytic leukemia. Leukemia. 2017;31:2815–23. 28555081 10.1038/leu.2017.164 PMC8370480

[10] Viny AD, Bowman RL, Liu Y, Lavallee VP, Eisman SE, Xiao W, et al. Cohesin Members Stag1 and Stag2 display distinct roles in chromatin accessibility and topological control of HSC self-renewal and differentiation. Cell Stem Cell. 2019;25:682–96.e8. 31495782 10.1016/j.stem.2019.08.003 PMC6842438

[11] Viny AD, Ott CJ, Spitzer B, Rivas M, Meydan C, Papalexi E, et al. Dose-dependent role of the cohesin complex in normal and malignant hematopoiesis. J Exp Med. 2015;212:1819–32. 26438361 10.1084/jem.20151317 PMC4612085

[12] Mazumdar C, Shen Y, Xavy S, Zhao F, Reinisch A, Li R, et al. Leukemia-associated cohesin mutants dominantly enforce stem cell programs and impair human hematopoietic progenitor differentiation. Cell Stem Cell. 2015;17:675–88. 26607380 10.1016/j.stem.2015.09.017 PMC4671831

[13] Mullenders J, Aranda-Orgilles B, Lhoumaud P, Keller M, Pae J, Wang K, et al. Cohesin loss alters adult hematopoietic stem cell homeostasis, leading to myeloproliferative neoplasms. J Exp Med. 2015;212:1833–50. 26438359 10.1084/jem.20151323 PMC4612095

[14] Tothova Z, Valton AL, Gorelov RA, Vallurupalli M, Krill-Burger JM, Holmes A, et al. Cohesin mutations alter DNA damage repair and chromatin structure and create therapeutic vulnerabilities in MDS/AML. JCI Insight. 2021;6:e142149. 33351783 10.1172/jci.insight.142149 PMC7934867

[15] Ochi Y, Kon A, Sakata T, Nakagawa MM, Nakazawa N, Kakuta M, et al. Combined Cohesin-RUNX1 deficiency synergistically perturbs chromatin looping and causes myelodysplastic syndromes. Cancer Discov. 2020;10:836–53. 32249213 10.1158/2159-8290.CD-19-0982 PMC7269820

[16] Wang T, Glover B, Hadwiger G, Miller CA, di Martino O, Welch JS. Smc3 is required for mouse embryonic and adult hematopoiesis. Exp Hematol. 2019;70:70–84. e76 30553776 10.1016/j.exphem.2018.11.008 PMC6639053

[17] Chen Z, Amro EM, Becker F, Holzer M, Rasa SMM, Njeru SN, et al. Cohesin-mediated NF-kappaB signaling limits hematopoietic stem cell self-renewal in aging and inflammation. J Exp Med. 2019;216:152–75. 30530755 10.1084/jem.20181505 PMC6314529

[18] Galeev R, Baudet A, Kumar P, Rundberg Nilsson A, Nilsson B, Soneji S, et al. Genome-wide RNAi screen identifies cohesin genes as modifiers of renewal and differentiation in human HSCs. Cell Rep. 2016;14:2988–3000. 26997282 10.1016/j.celrep.2016.02.082 PMC7616965

[19] Tothova Z, Krill-Burger JM, Popova KD, Landers CC, Sievers QL, Yudovich D, et al. Multiplex CRISPR/Cas9-based genome editing in human hematopoietic stem cells models clonal hematopoiesis and myeloid neoplasia. Cell Stem Cell. 2017;21:547–55.e548. 28985529 10.1016/j.stem.2017.07.015 PMC5679060

[20] Papaemmanuil E, Gerstung M, Bullinger L, Gaidzik VI, Paschka P, Roberts ND, et al. Genomic classification and prognosis in acute myeloid leukemia. N Engl J Med. 2016;374:2209–21. 27276561 10.1056/NEJMoa1516192 PMC4979995

[21] Lindsley RC, Mar BG, Mazzola E, Grauman PV, Shareef S, Allen SL, et al. Acute myeloid leukemia ontogeny is defined by distinct somatic mutations. Blood. 2015;125:1367–76. 25550361 10.1182/blood-2014-11-610543 PMC4342352

[22] Dohner H, Wei AH, Appelbaum FR, Craddock C, DiNardo CD, Dombret H, et al. Diagnosis and management of AML in adults: 2022 recommendations from an international expert panel on behalf of the ELN. Blood. 2022;140:1345–77. 35797463 10.1182/blood.2022016867

[23] Thol F, Bollin R, Gehlhaar M, Walter C, Dugas M, Suchanek KJ, et al. Mutations in the cohesin complex in acute myeloid leukemia: clinical and prognostic implications. Blood. 2014;123:914–20. 24335498 10.1182/blood-2013-07-518746

[24] Eckardt JN, Stasik S, Rollig C, Sauer T, Scholl S, Hochhaus A, et al. Alterations of cohesin complex genes in acute myeloid leukemia: differential co-mutations, clinical presentation and impact on outcome. Blood Cancer J. 2023;13:18. 36693840 10.1038/s41408-023-00790-1 PMC9873811

[25] Arber DA, Orazi A, Hasserjian R, Thiele J, Borowitz MJ, Le Beau MM, et al. The 2016 revision to the World Health Organization classification of myeloid neoplasms and acute leukemia. Blood. 2016;127:2391–405. 27069254 10.1182/blood-2016-03-643544

[26] Kluk MJ, Lindsley RC, Aster JC, Lindeman NI, Szeto D, Hall D, et al. Validation and implementation of a custom next-generation sequencing clinical assay for hematologic malignancies. J Mol Diagn. 2016;18:507–15. 27339098 10.1016/j.jmoldx.2016.02.003 PMC5707186

[27] Haferlach T, Schoch C, Loffler H, Gassmann W, Kern W, Schnittger S, et al. Morphologic dysplasia in de novo acute myeloid leukemia (AML) is related to unfavorable cytogenetics but has no independent prognostic relevance under the conditions of intensive induction therapy: results of a multiparameter analysis from the German AML Cooperative Group studies. J Clin Oncol. 2003;21:256–65. 12525517 10.1200/JCO.2003.08.005

[28] Schoch C, Schnittger S, Bursch S, Gerstner D, Hochhaus A, Berger U, et al. Comparison of chromosome banding analysis, interphase- and hypermetaphase-FISH, qualitative and quantitative PCR for diagnosis and for follow-up in chronic myeloid leukemia: a study on 350 cases. Leukemia. 2002;16:53–59. 11840263 10.1038/sj.leu.2402329

[29] Kern W, Voskova D, Schoch C, Hiddemann W, Schnittger S, Haferlach T. Determination of relapse risk based on assessment of minimal residual disease during complete remission by multiparameter flow cytometry in unselected patients with acute myeloid leukemia. Blood. 2004;104:3078–85. 15284114 10.1182/blood-2004-03-1036

[30] Chakravarty D, Gao J, Phillips SM, Kundra R, Zhang H, Wang J, et al. OncoKB: A Precision Oncology Knowledge Base. JCO Precis Oncol. 2017;1:1–16. 10.1200/PO.17.00011 PMC5586540 28890946

[31] Tate JG, Bamford S, Jubb HC, Sondka Z, Beare DM, Bindal N, et al. COSMIC: the Catalogue Of Somatic Mutations In Cancer. Nucleic Acids Res. 2019;47:D941–47. 30371878 10.1093/nar/gky1015 PMC6323903

[32] Baer C, Pohlkamp C, Haferlach C, Kern W, Haferlach T. Molecular patterns in cytopenia patients with or without evidence of myeloid neoplasm-a comparison of 756 cases. Leukemia. 2018;32:2295–8. 29725031 10.1038/s41375-018-0119-8

[33] Meggendorfer M, Haferlach C, Kern W, Haferlach T. Molecular analysis of myelodysplastic syndrome with isolated deletion of the long arm of chromosome 5 reveals a specific spectrum of molecular mutations with prognostic impact: a study on 123 patients and 27 genes. Haematologica. 2017;102:1502–10. 28642303 10.3324/haematol.2017.166173 PMC5685225

[34] Huber S, Baer C, Hutter S, Dicker F, Meggendorfer M, Pohlkamp C, et al. AML classification in the year 2023: How to avoid a Babylonian confusion of languages. Leukemia. 2023;37:1413–20. 37120689 10.1038/s41375-023-01909-w PMC10317829

[35] Huber S, Haferlach T, Meggendorfer M, Hutter S, Hoermann G, Baer C, et al. SF3B1 mutated MDS: Blast count, genetic co-abnormalities and their impact on classification and prognosis. Leukemia. 2022;36:2894–902. 36261576 10.1038/s41375-022-01728-5 PMC9712089

[36] Tyner JW, Tognon CE, Bottomly D, Wilmot B, Kurtz SE, Savage SL, et al. Functional genomic landscape of acute myeloid leukaemia. Nature. 2018;562:526–31. 30333627 10.1038/s41586-018-0623-z PMC6280667

[37] Zhou Y, Zhou B, Pache L, Chang M, Khodabakhshi AH, Tanaseichuk O, et al. Metascape provides a biologist-oriented resource for the analysis of systems-level datasets. Nat Commun. 2019;10:1523. 30944313 10.1038/s41467-019-09234-6 PMC6447622

[38] Bernard E, Nannya Y, Hasserjian RP, Devlin SM, Tuechler H, Medina-Martinez JS, et al. Implications of TP53 allelic state for genome stability, clinical presentation and outcomes in myelodysplastic syndromes. Nat Med. 2020;26:1549–56. 32747829 10.1038/s41591-020-1008-z PMC8381722

[39] Weinberg OK, Siddon A, Madanat YF, Gagan J, Arber DA, Dal Cin P, et al. TP53 mutation defines a unique subgroup within complex karyotype de novo and therapy-related MDS/AML. Blood Adv. 2022;6:2847–53. 35073573 10.1182/bloodadvances.2021006239 PMC9092405

[40] Arber DA, Orazi A, Hasserjian RP, Borowitz MJ, Calvo KR, Kvasnicka HM, et al. International consensus classification of myeloid neoplasms and acute leukemias: integrating morphologic, clinical, and genomic data. Blood. 2022;140:1200–28. 35767897 10.1182/blood.2022015850 PMC9479031

[41] Tazi Y, Arango-Ossa JE, Zhou Y, Bernard E, Thomas I, Gilkes A, et al. Unified classification and risk-stratification in acute myeloid leukemia. Nat Commun. 2022;13:4622. 35941135 10.1038/s41467-022-32103-8 PMC9360033

[42] Minchell NE, Keszthelyi A, Baxter J. Cohesin causes replicative DNA damage by trapping DNA topological stress. Mol Cell. 2020;78:739–51.e738. 32259483 10.1016/j.molcel.2020.03.013 PMC7242899

[43] Arnould C, Rocher V, Finoux AL, Clouaire T, Li K, Zhou F, et al. Loop extrusion as a mechanism for formation of DNA damage repair foci. Nature. 2021;590:660–5. 33597753 10.1038/s41586-021-03193-z PMC7116834

[44] Mandal PK, Ferreira LM, Collins R, Meissner TB, Boutwell CL, Friesen M, et al. Efficient ablation of genes in human hematopoietic stem and effector cells using CRISPR/Cas9. Cell Stem Cell. 2014;15:643–52. 25517468 10.1016/j.stem.2014.10.004 PMC4269831

[45] Su XA, Ma D, Parsons JV, Replogle JM, Amatruda JF, Whittaker CA, et al. RAD21 is a driver of chromosome 8 gain in Ewing sarcoma to mitigate replication stress. Genes Dev. 2021;35:556–72. 33766983 10.1101/gad.345454.120 PMC8015718

