# Supplementary material for: Subunit-specific analysis of cohesin-mutant myeloid malignancies reveals distinct ontogeny and outcomes
Source: Leukemia. 2024 Jul 20;38(9):1992–2002. doi: 10.1038/s41375-024-02347-y (PMC11347381; doi:10.1038/s41375-024-02347-y)
Supplement: Supplementary file 1 — Supplementary Figures [file 41375_2024_2347_MOESM1_ESM.pdf]

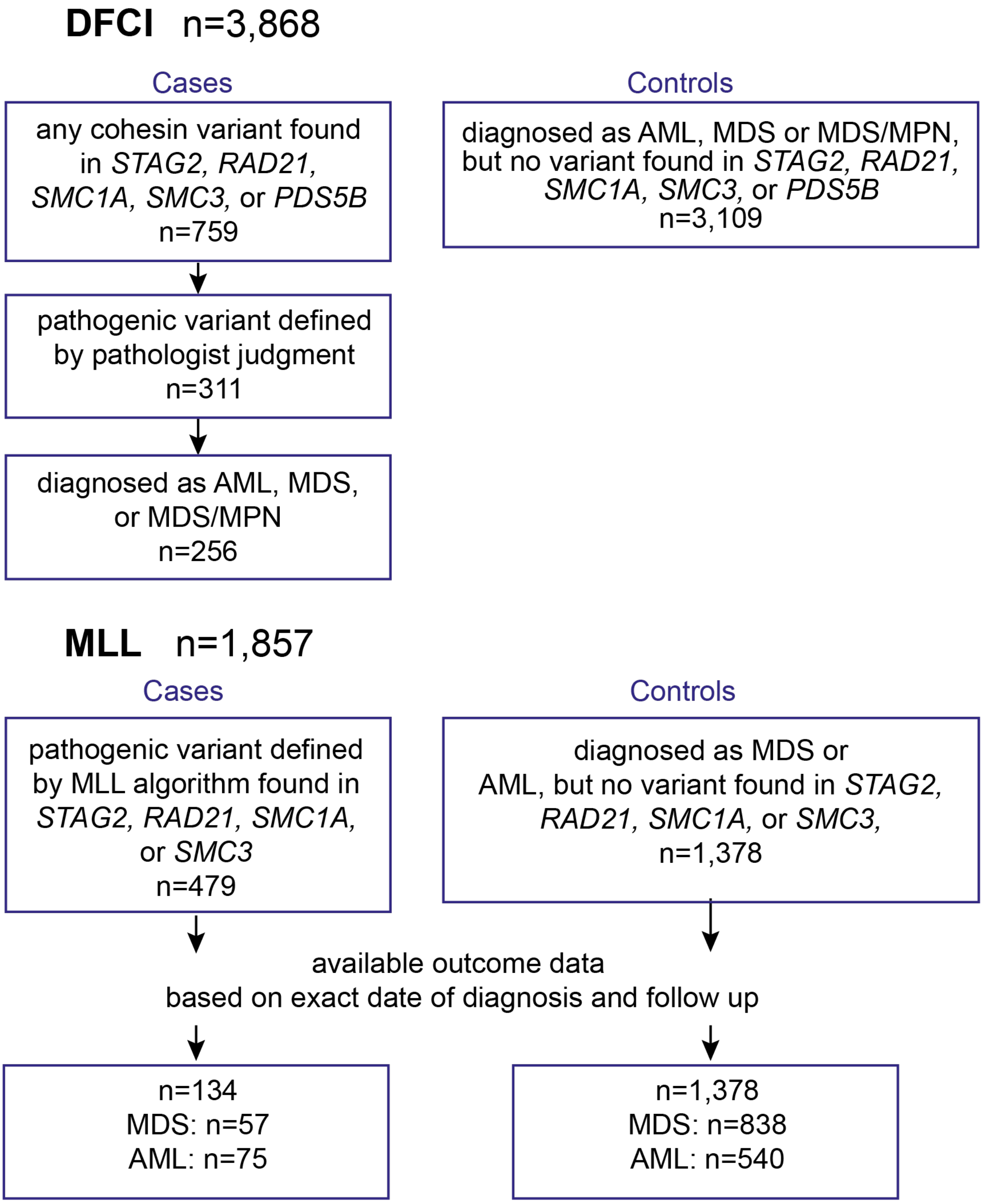

Supplementary Figure 1: CONSORT diagram of patient flow in this study

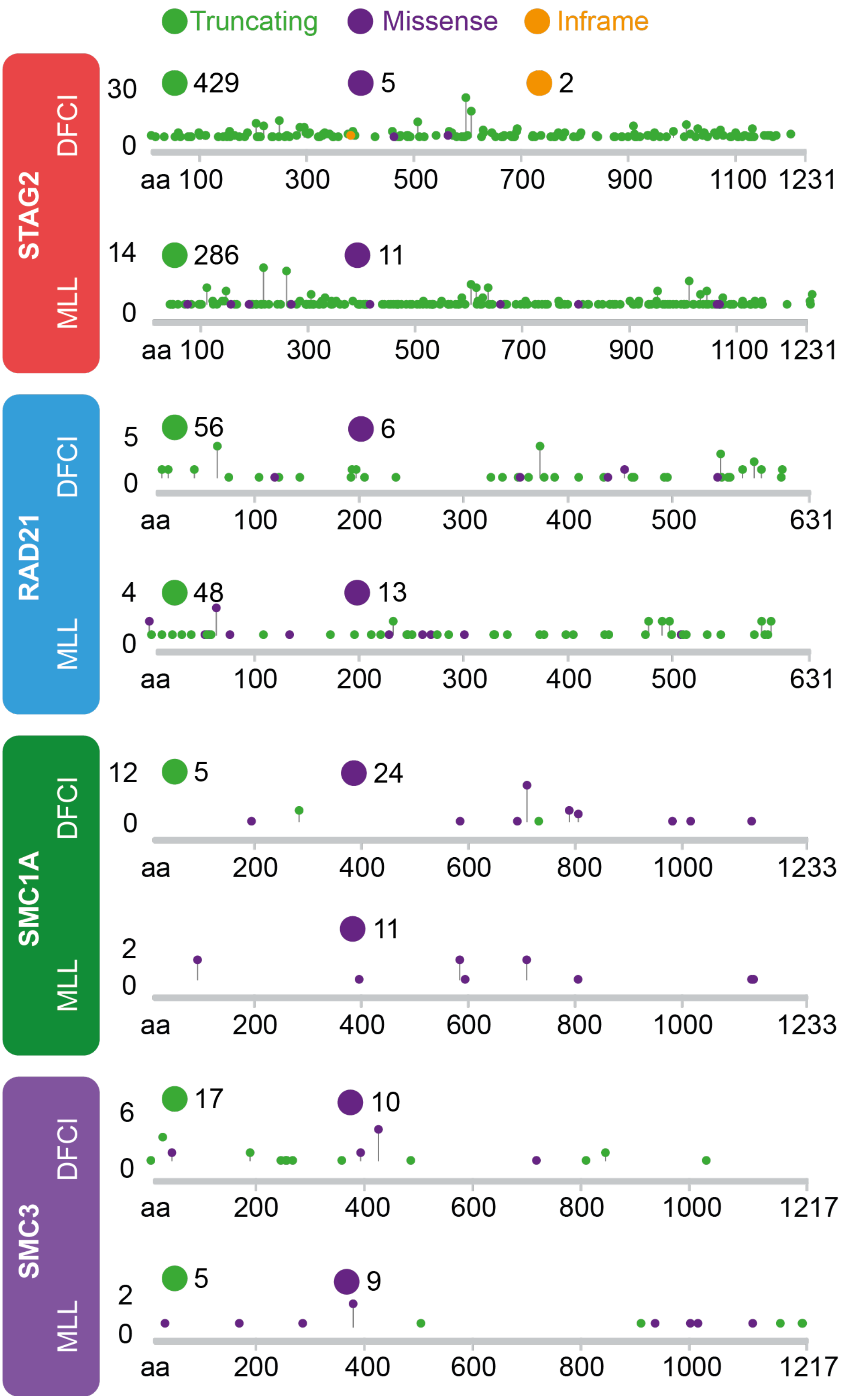

Supplementary Figure 2: Molecular characterization of cohesin mutations in hematologic malignancies separated by cohort.

Lollipop plot panel of cohesin mutations separated by cohort and cohesin gene. DFCI = Dana-Farber Cancer Institute cohort, MLL = Munich Leukemia Laboratory cohort.

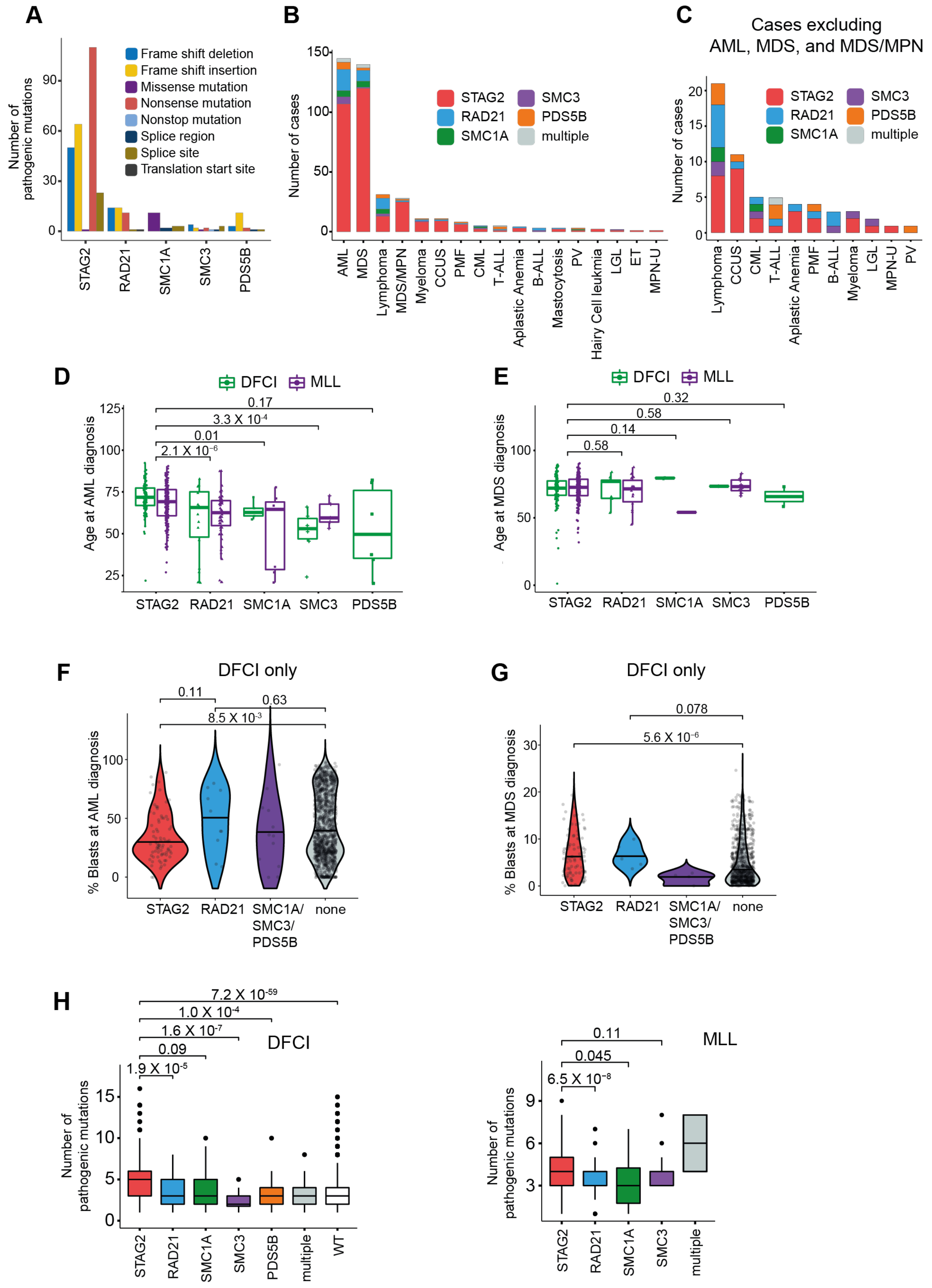

Supplementary Figure 3: Clinical characteristics of cohesin-mutant patients separated by cohort
(A) Bar plot of the total number of pathogenic mutations in the DFCI cohort. **(B)** Bar plot of the total number of cases per diagnosis for patients with a pathogenic cohesin mutation in the DFCI cohort. **(C)** Bar plot of the total number of patients with a pathogenic cohesin mutation, in whom no diagnosis of AML, MDS, or MDS/MPN was made. Multiple diagnoses can be counted per single patient, n=55. **(D-E)** Box plots of age at diagnosis for MDS or AML stratified by cohesin mutation. Benjamini Hochberg adjusted p values for specific mutations are shown. **(F-G)** Violin plots of %blasts in diagnostic bone marrow specimens for patients diagnosed with AML or MDS in the DFCI cohort, stratified by cohesin mutation and compared to the cohesin WT cohort. Benjamini Hochberg adjusted p values are shown. **(H)** Box plot of total number of pathogenic mutations determined by myeloid target capture sequencing for MDS and AML patients stratified by cohesin mutation status, separated by DFCI and MLL cohorts.

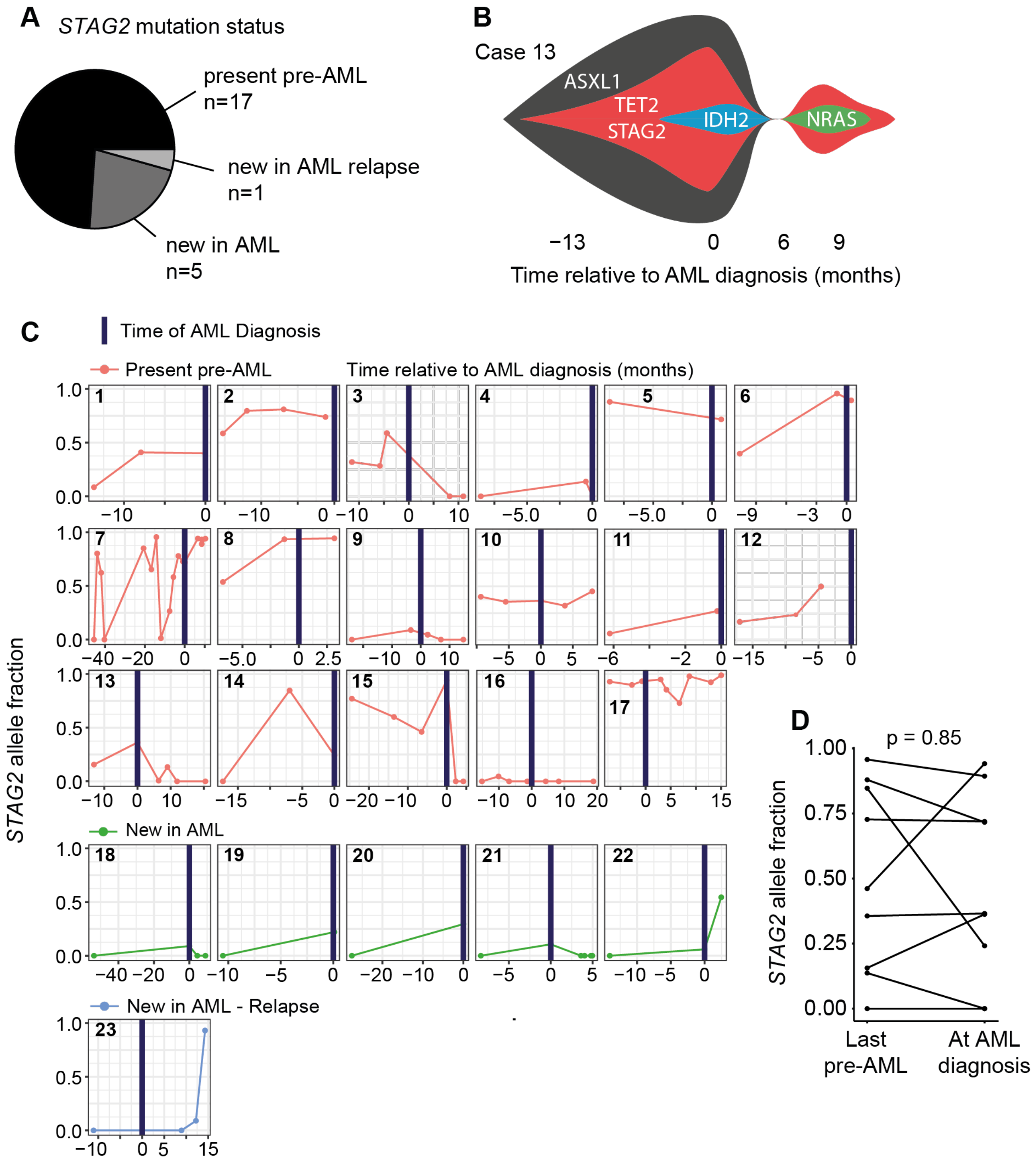

Supplementary Figure 4: Acquisition of *STAG2* mutations relative to the onset of AML
(A) Pie chart of *STAG2* mutation detection relative to AML diagnosis. **(B)** Fish plot for clonal evolution of a representative *STAG2*-mutant case, with *STAG2* mutation preceding the AML diagnosis. Clone sizes are extrapolated from variant allele fraction (VAF) as previously reported[42]. **(C)** VAF quantification for *STAG2*-mutant patients with at least 1 available mutational panel testing prior to AML diagnosis. Each box represents a single patient. Patients were grouped according to the presence or absence of the *STAG2* mutation prior to AML diagnosis using a *STAG2* VAF cutoff of 0.01. Thick black line indicates timepoint of AML diagnosis. **(D)** VAF quantification for paired mutational assessment pre-AML and at the time of AML diagnosis. Two sided paired t-test was used to determine statistical significance.

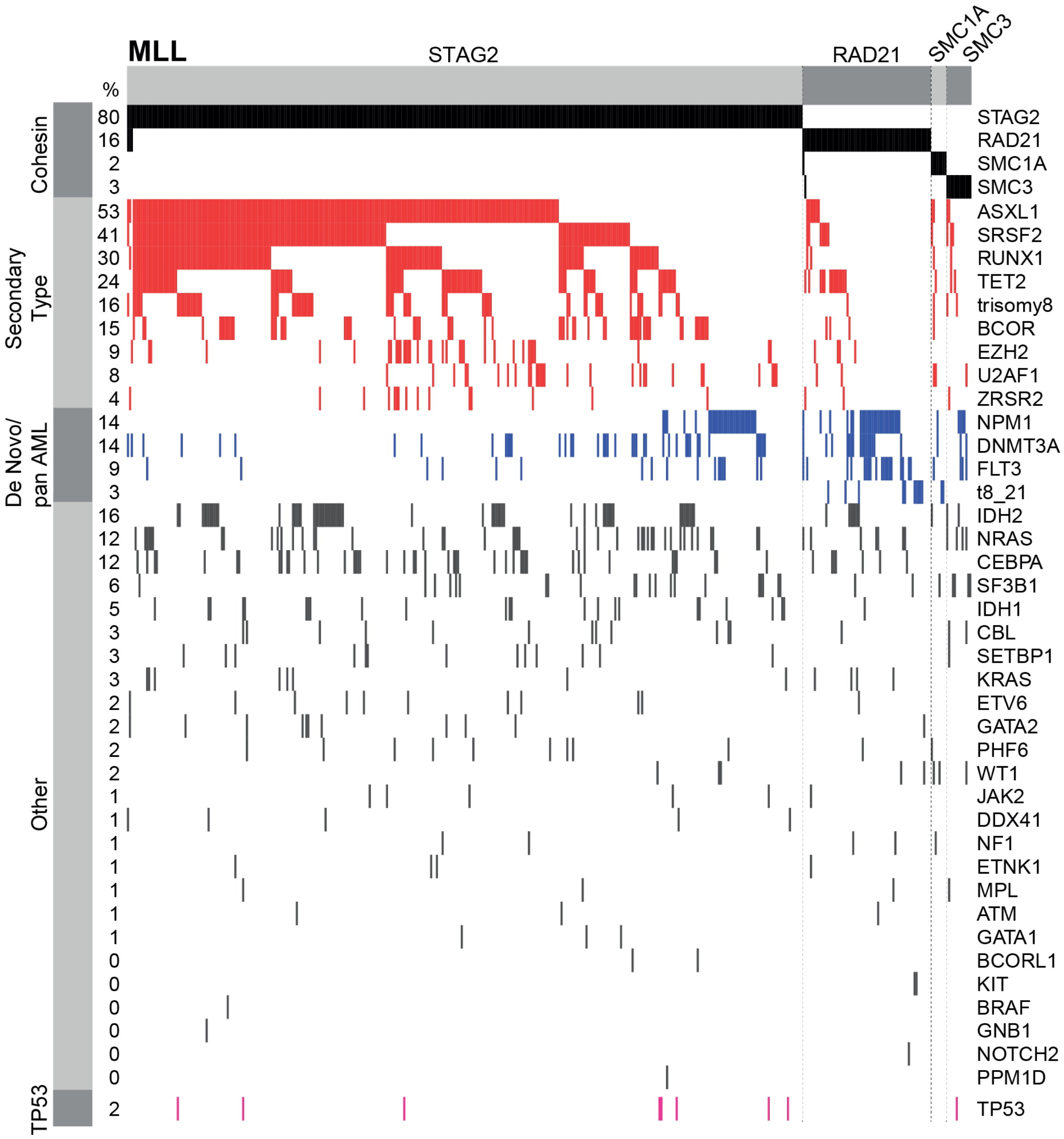

Supplementary Figure 5: Co-mutational plot for different cohesin mutations in the MLL cohort
Oncoprint for MDS, AML, and MDS/MPN patients with *STAG2, RAD21, SMC3,* and *SMC1A* mutations sorted by co-mutational pattern.

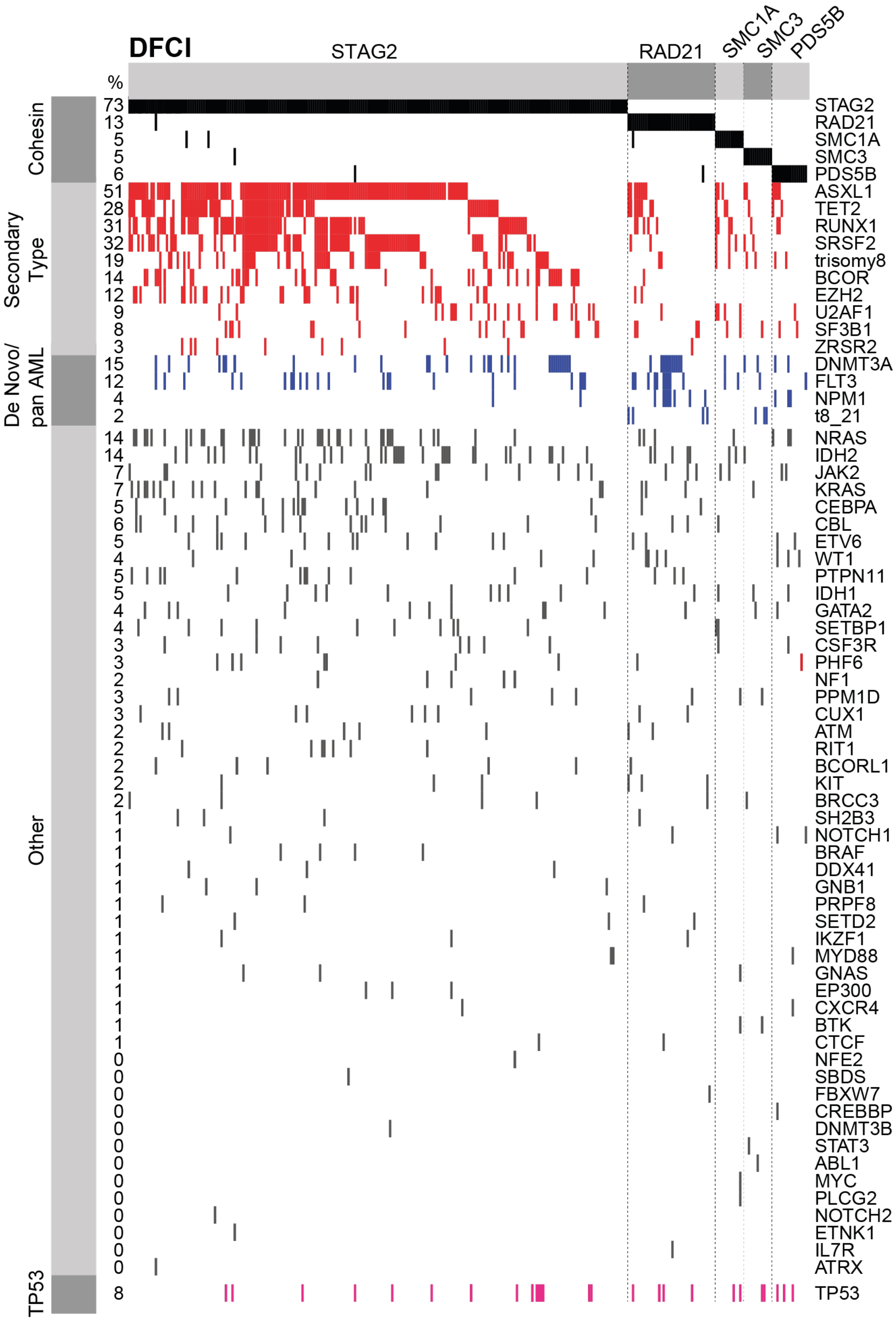

Supplementary Figure 6: Co-mutational plot for different cohesin mutations in the DFCI cohort
Oncoprint for MDS, AML, and MDS/MPN patients with *STAG2, RAD21, SMC3, SMC1A,* and *PDS5B* mutations sorted by co-mutational pattern.

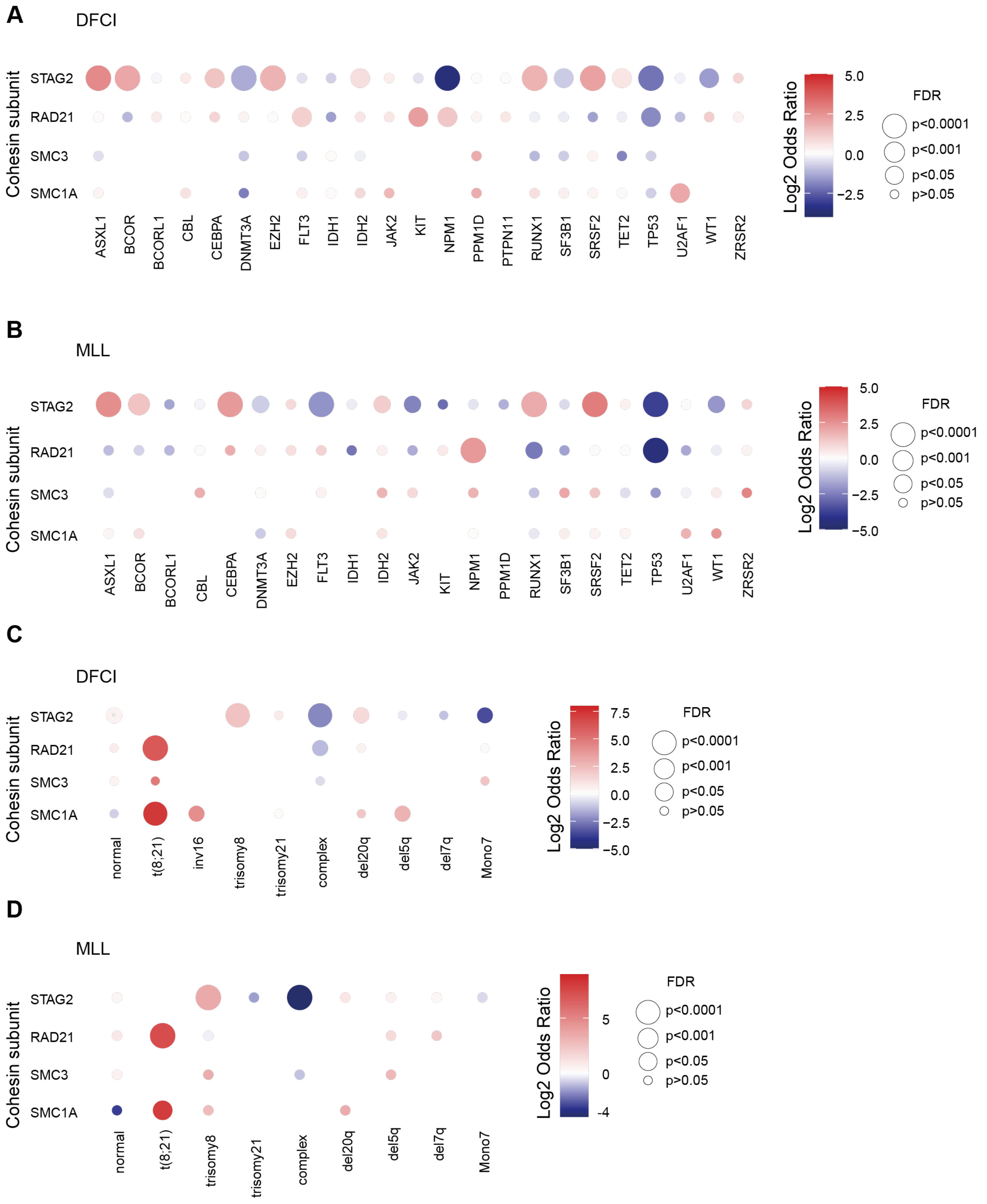

Supplementary Figure 7: Mutations in different cohesin subunits display unique mutational and chromosomal abnormality characteristics separated by cohort

(A-B) Balloon plot for relative enrichment of co-occurrence of cohesin subunit mutations with other myeloid driver mutations and **(C-D)** chromosomal aberrations, separated by cohort. Cohesin WT cohort was used as a reference cohort to calculate enrichment, which is indicated as log2 odds ratio (OR). Combinations with q<0.05 or 5% mutational frequency in the total cohort are shown.

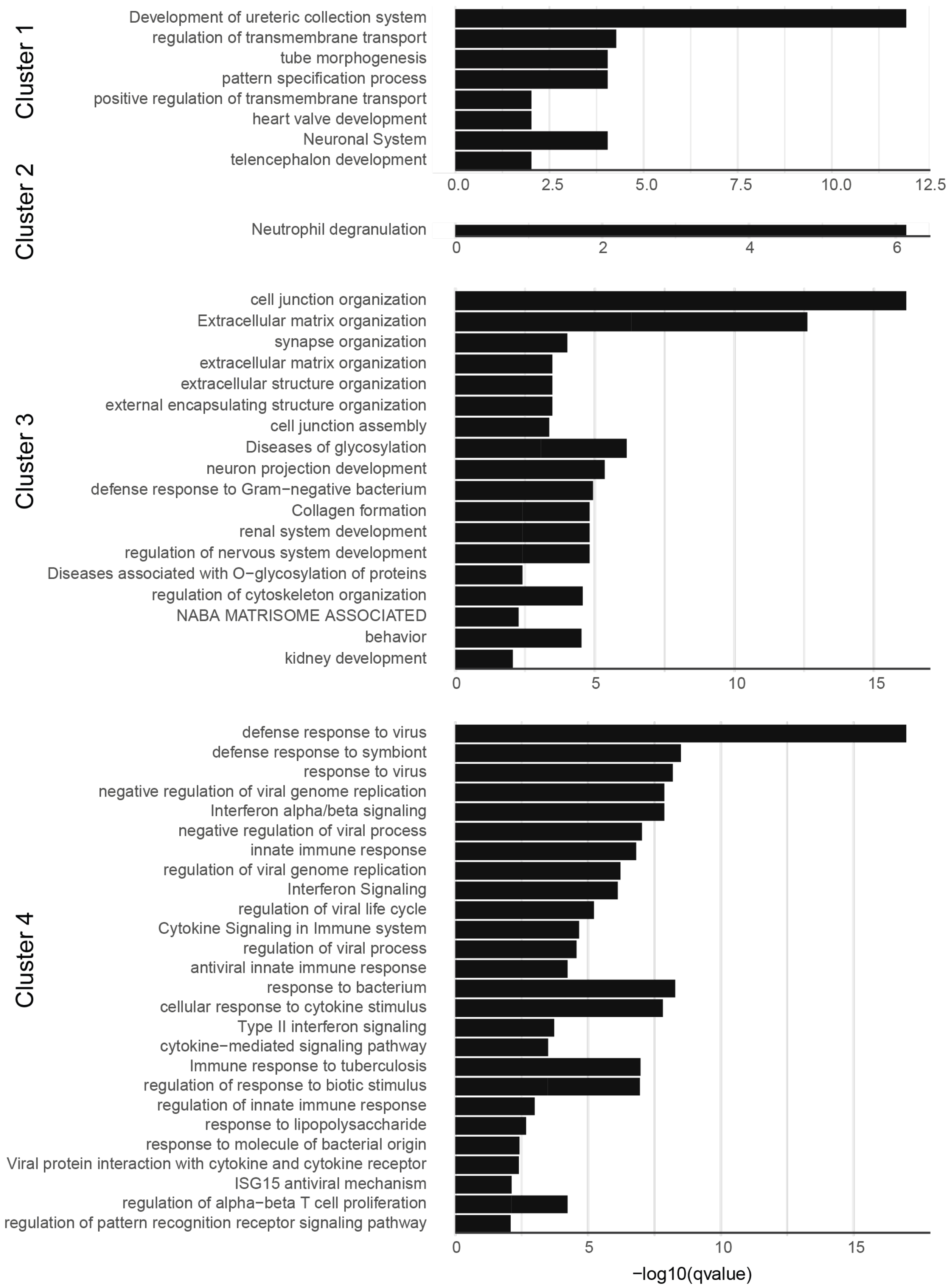

Supplementary Figure 8: Gene set enrichment analysis of cohesin-mutant AML RNA-Seq data in the BeatAML[36] cohort.
Gene sets with an FDR<0.01 were plotted for each cluster derived from unsupervised k-means clustering from Fig. 3C. Clusters 1-4 include 194, 401, 463, and 270 genes, respectively.

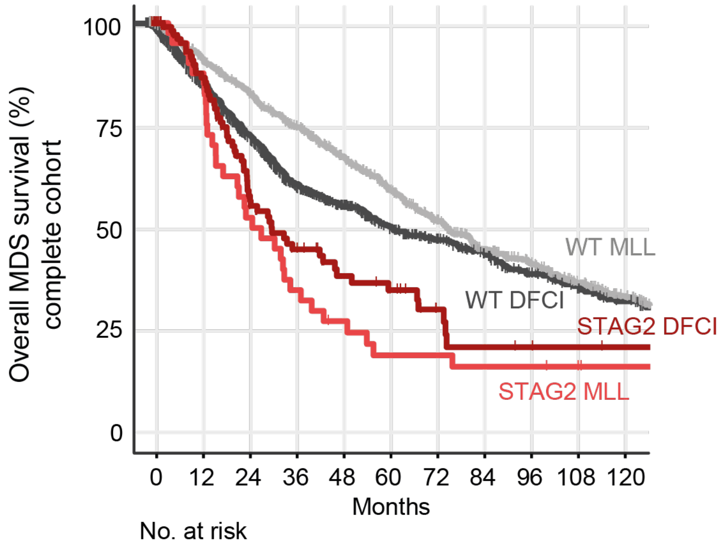

No. at risk

	0	12	24	36	48	60	72	84	96	108	120
**WT DFCI**	1023	738	542	381	292	213	170	136	103	82	66
**WT MLL**	774	675	597	514	432	354	289	228	192	156	122
**STAG2 DFCI**	119	82	53	33	23	20	12	7	6	5	4
**STAG2 MLL**	41	36	21	14	10	7	7	6	6	5	3

	HR	95% CI	p-val
**STAG2 DFCI**	1.14	0.87, 1.48	0.3
**STAG2 MLL**	2.12	1.5, 3.01	<0.001

Supplementary Figure 9: MDS-related outcomes separated by cohort
Survival analysis using the Kaplan-Meier method and log rank test for overall MDS survival stratified for *STAG2*-mutant and cohesin-WT status in the DFCI and MLL cohorts.

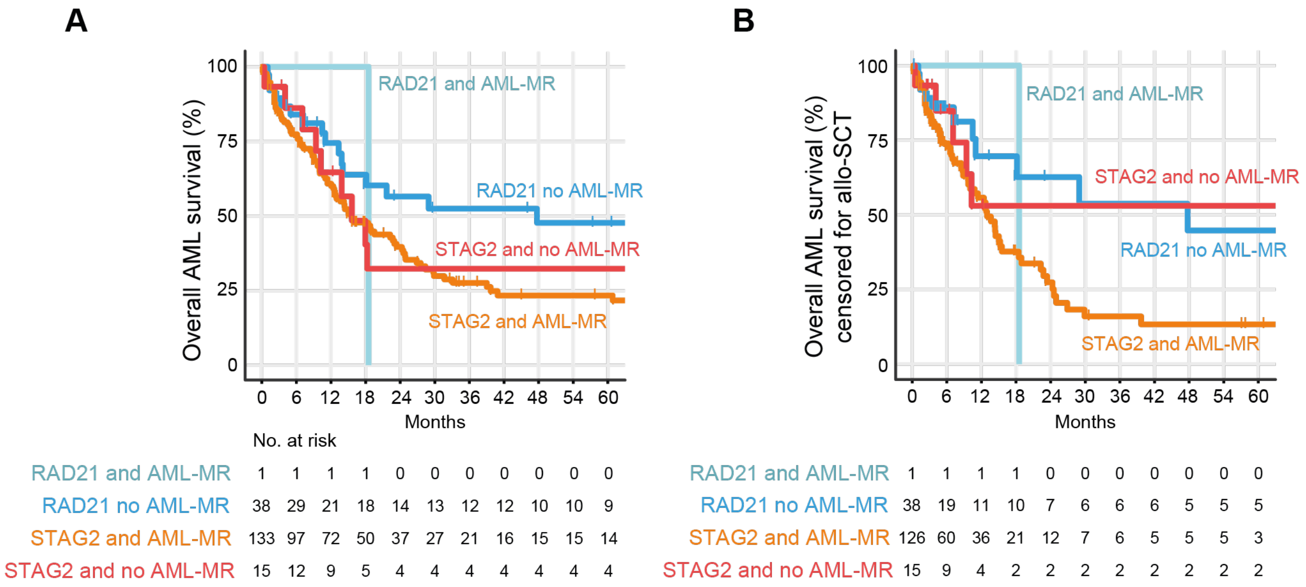

Supplementary Figure 10: AML-related outcomes in the combined dataset
(A) Survival analysis using the Kaplan-Meier method for overall AML survival stratified for cohesin mutational status and WHO2022 diagnosis. **(B)** Subgroup analysis censored at the time of allogeneic stem cell transplantation (allo-SCT).

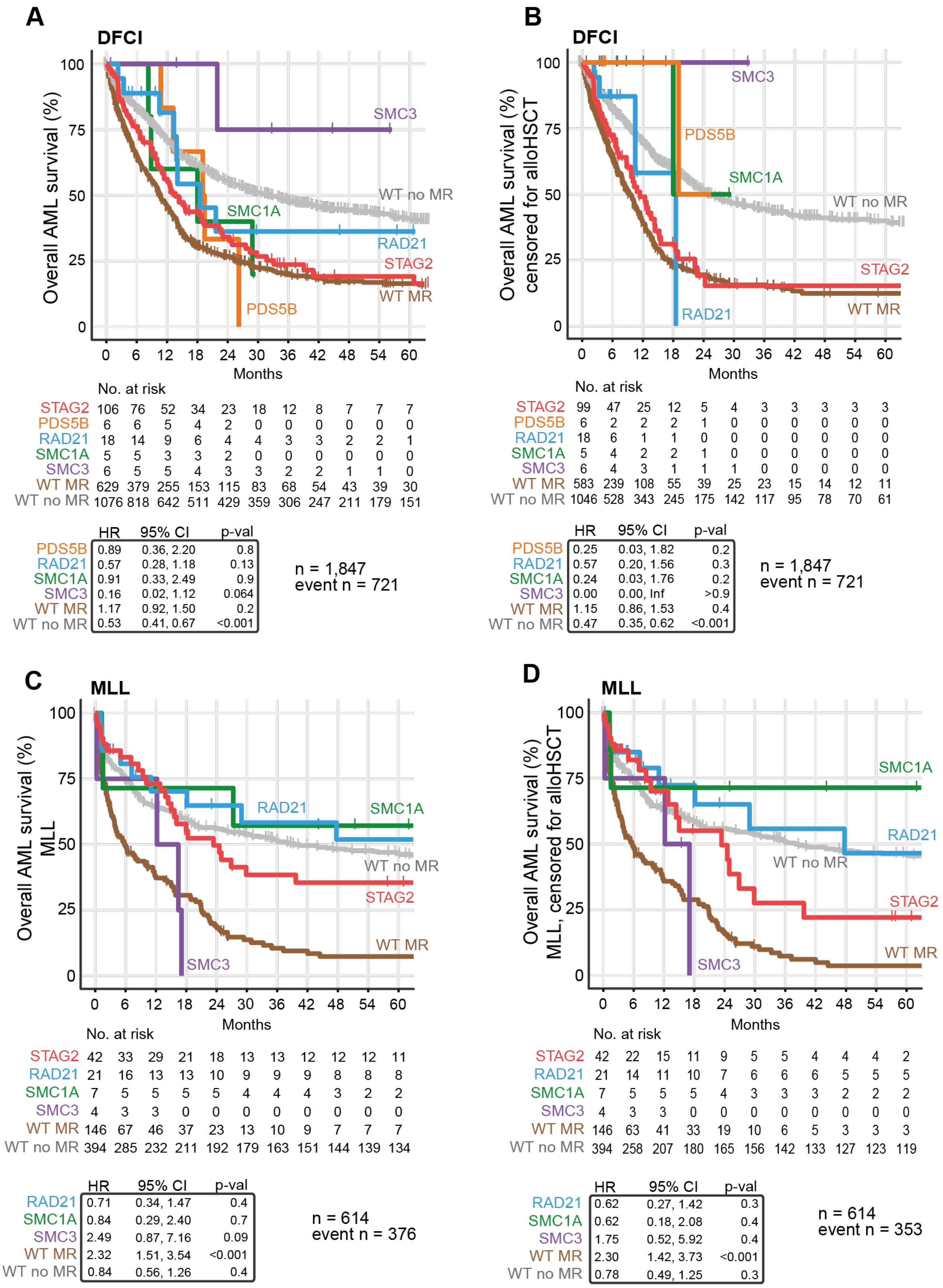

Supplementary Figure 11: AML-related outcomes separated by cohort
(A-B) DFCI cohort: **(A)** Survival analysis using the Kaplan-Meier method and log-rank test for overall AML survival stratified for cohesin mutational status and cohesin-WT cases separated by AML-MR and AML-non-MR. **(B)** Overall AML survival for above subgroups censored at the time of allogeneic stem cell transplantation. Table indicates hazard ratio from univariate model. **(C-D)** Same as **(A-B)** above for the MLL cohort. HR=Hazard ratio, 95% CI = 95% Confidence interval.

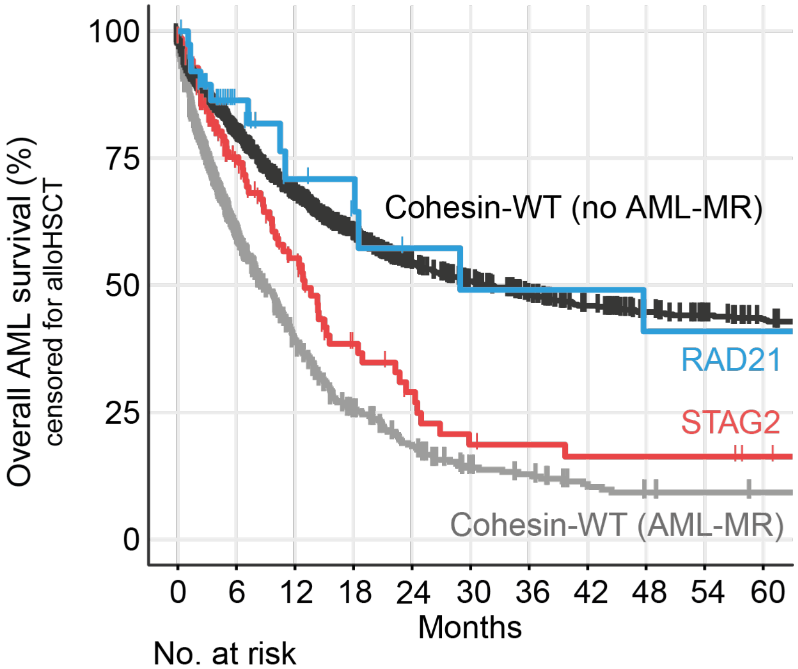

No. at risk

	0	6	12	18	24	30	36	42	48	54	60
STAG2	141	69	40	23	14	9	8	7	7	7	5
RAD21	39	20	12	11	7	6	6	6	5	5	5
Cohesin-WT (AML-MR)	729	302	149	88	58	35	29	20	17	15	14
Cohesin-WT (no AML-MR)	1440	786	550	425	340	298	259	228	205	193	180

STAG2 vs. AML-noMR: HR: 0.54 p <0.001
STAG2 vs. RAD21: HR: 0.48 p =0.018

Supplementary Figure 12: AML survival censored for allogeneic stem cell transplant
AML overall survival stratified by cohesin subunit mutation status, and cohesin-WT group by AML MR or AML-non-MR, censored at the time of first of allogeneic stem cell transplantation. HR=Hazard ratio. Statistical significance was determined using the log rank-test.
