# Supplementary material for: Subunit-specific analysis of cohesin-mutant myeloid malignancies reveals distinct ontogeny and outcomes
Source: Leukemia. 2024 Jul 20;38(9):1992–2002. doi: 10.1038/s41375-024-02347-y (PMC11347381; doi:10.1038/s41375-024-02347-y)
Supplement: Supplementary file 4 — Supplementary Table 3 [file 41375_2024_2347_MOESM4_ESM.pdf]

Supplementary Table 3. **Patient and disease characteristics of different cohesin complex mutations in MDS, MDS/MPN, AML patients by cohort**

DFCI Cohort

Characteristic	N	STAG2<br>198	RAD21<br>26	SMC1A<br>10	SMC3<br>7	PDS5B<br>9	multiple<br>6	Cohesin WT<br>3,109	Overall<br>3,365
**Sex** *n/N (%)*	**3,365**								
Female		70/224 (31%)	9/38 (24%)	4/13 (31%)	5/13 (38%)	6/16 (38%)	3/7 (43%)	1,197/3,109 (39%)	1,294/3,420 (38%)
Male		154/224 (69%)	29/38 (76%)	9/13 (69%)	8/13 (62%)	10/16 (62%)	4/7 (57%)	1,912/3,109 (61%)	2,126/3,420 (62%)
...									
**Age at...** *Median (Range)*									
MDS diagnosis	**1,446**	72 (1, 89)	77 (54, 84)	80 (79, 80)	73 (73, 73)	66 (58, 73)	80 (77, 88)	69 (14, 93)	69 (1, 93)
AML diagnosis	**1,852**	72 (22, 92)	66 (21, 83)	63 (59, 72)	53 (24, 66)	50 (20, 82)	73 (50, 76)	65 (1, 93)	65 (1, 93)
**MDS WHO2022, n / N (%)**	**1,446**	120 / 1,446 (8%)	9 / 1,446 (0.6%)	9 / 1,446 (0.3%)	1 / 1,446 (>0.1%)	1 / 1,446 (0.1%)	1 / 1,446 (0.2%)		
within subunit		**120 / 198 (61%)**	**9 / 26 (35%)**	**5 / 10 (50%)**	**1 / 7 (14%)**	**2 / 9 (22%)**	**3 / 6 (50%)**	**1,306 / 3109 (42%)**	**1,446 / 3,365 (43%)**
MDS-5q		1 / 120 (0.8%)	0 / 9 (0%)	0 / 5 (0%)	0 / 1 (0%)	0 / 2 (0%)	0 / 3 (0%)	62 / 1,306 (4.7%)	63 / 1,446 (4.4%)
MDS-biTP53		0 / 120 (0%)	0 / 9 (0%)	0 / 5 (0%)	0 / 1 (0%)	0 / 2 (0%)	0 / 3 (0%)	15 / 1,306 (1.1%)	15 / 1,446 (1.0%)
MDS-IB1		40 / 120 (33%)	2 / 9 (22%)	0 / 5 (0%)	0 / 1 (0%)	1 / 2 (50%)	1 / 3 (33%)	196 / 1,306 (15%)	240 / 1,446 (17%)
MDS-IB2		39 / 120 (32%)	3 / 9 (33%)	0 / 5 (0%)	0 / 1 (0%)	0 / 2 (0%)	1 / 3 (33%)	245 / 1,306 (19%)	288 / 1,446 (20%)
MDS-LB		36 / 120 (30%)	3 / 9 (33%)	4 / 5 (80%)	1 / 1 (100%)	1 / 2 (50%)	1 / 3 (33%)	647 / 1,306 (50%)	693 / 1,446 (48%)
MDS-SF3B1		4 / 120 (3.3%)	1 / 9 (11%)	1 / 5 (20%)	0 / 1 (0%)	0 / 2 (0%)	0 / 3 (0%)	141 / 1,306 (11%)	147 / 1,446 (10%)
**AML WHO2022, n / N (%)**	**1,852**	107 / 1,852 (6%)	18 / 1,852 (1%)	5 / 1,852 (0.2%)	6 / 1,852 (0.3%)	6 / 1,852 (0.3%)	3 / 1,852 (0.2%)		
within subunit		**107 / 198 (54%)**	**18 / 26 (69%)**	**5 / 10 (50%)**	**6 / 7 (85%)**	**6 / 9 (67%)**	**3 / 6 (50%)**	**1,707 / 3109 (55%)**	**1,852 / 3,365 (55%)**
Acute myeloid leukemia with BCR-ABL1		0 / 107 (0%)	0 / 18 (0%)	0 / 5 (0%)	0 / 6 (0%)	0 / 6 (0%)	0 / 3 (0%)	5 / 1,707 (0.3%)	5 / 1,852 (0.3%)
AML by differentiation		0 / 107 (0%)	10 / 18 (56%)	2 / 5 (40%)	1 / 6 (17%)	3 / 6 (50%)	1 / 3 (33%)	655 / 1,707 (38%)	672 / 1,852 (36%)
AML with biallelic CEBPA		1 / 107 (0.9%)	0 / 18 (0%)	0 / 5 (0%)	0 / 6 (0%)	0 / 6 (0%)	0 / 3 (0%)	12 / 1,707 (0.7%)	13 / 1,852 (0.7%)
AML with CBFB-MYH11		0 / 107 (0%)	0 / 18 (0%)	1 / 5 (20%)	1 / 6 (17%)	0 / 6 (0%)	0 / 3 (0%)	48 / 1,707 (2.8%)	50 / 1,852 (2.7%)
AML with DEK-NUP214		0 / 107 (0%)	0 / 18 (0%)	0 / 5 (0%)	0 / 6 (0%)	0 / 6 (0%)	0 / 3 (0%)	3 / 1,707 (0.2%)	3 / 1,852 (0.2%)
AML with GATA2, MECOM		0 / 107 (0%)	0 / 18 (0%)	0 / 5 (0%)	0 / 6 (0%)	0 / 6 (0%)	0 / 3 (0%)	11 / 1,707 (0.6%)	11 / 1,852 (0.6%)
AML with KMT2A-MLLT3		0 / 107 (0%)	0 / 18 (0%)	1 / 5 (20%)	0 / 6 (0%)	0 / 6 (0%)	0 / 3 (0%)	12 / 1,707 (0.7%)	13 / 1,852 (0.7%)
AML with NPM1		3 / 107 (2.8%)	5 / 18 (28%)	1 / 5 (20%)	0 / 6 (0%)	2 / 6 (33%)	0 / 3 (0%)	206 / 1,707 (12%)	217 / 1,852 (12%)
AML with PML-RARA		0 / 107 (0%)	0 / 18 (0%)	0 / 5 (0%)	1 / 6 (17%)	0 / 6 (0%)	0 / 3 (0%)	78 / 1,707 (4.6%)	79 / 1,852 (4.3%)
AML with RUNX1-RUNX1T1		1 / 107 (0.9%)	2 / 18 (11%)	0 / 5 (0%)	1 / 6 (17%)	0 / 6 (0%)	2 / 3 (67%)	29 / 1,707 (1.7%)	35 / 1,852 (1.9%)
AML-MR		102 / 107 (95%)	1 / 18 (5.6%)	0 / 5 (0%)	2 / 6 (33%)	1 / 6 (17%)	0 / 3 (0%)	629 / 1,707 (37%)	735 / 1,852 (40%)
Myeloid Sarcoma		0 / 107 (0%)	0 / 18 (0%)	0 / 5 (0%)	0 / 6 (0%)	0 / 6 (0%)	0 / 3 (0%)	19 / 1,707 (1.1%)	19 / 1,852 (1.0%)
**MDS/MPN WHO2022** *n/N (%)*	**466**	21 / 466 (5%)	1 / 466 (0.2%)	0 / 466 (0%)	0 / 466 (0%)	1 / 466 (0.2%)	1 / 466 (0.2%)		
within subunit		**21 / 198 (11%)**	**1 / 26 (4%)**	**0 / 10 (0%)**	**0 / 7 (0%)**	**1 / 9 (11%)**	**1 / 6 (17%)**	**442 / 3,109 (14%)**	**466 / 3,365 (14%)**
aCML/BCR-ABL1 negative		1/21 (4.8%)	0/1 (0%)	0/0 (NA%)	0/0 (NA%)	0/1 (0%)	0/1 (0%)	30/442 (6.8%)	31/466 (6.7%)
CMML		13/21 (62%)	0/1 (0%)	0/0 (NA%)	0/0 (NA%)	1/1 (100%)	0/1 (0%)	289/442 (65%)	303/466 (65%)
MDS/MPN-u		5/21 (24%)	1/1 (100%)	0/0 (NA%)	0/0 (NA%)	0/1 (0%)	0/1 (0%)	64/442 (14%)	70/466 (15%)
MDS/MPN with ring sideroblasts and thrombocytosis		0/21 (0%)	0/1 (0%)	0/0 (NA%)	0/0 (NA%)	0/1 (0%)	0/1 (0%)	44/442 (10.0%)	44/466 (9.4%)
Other		2/21 (9.5%)	0/1 (0%)	0/0 (NA%)	0/0 (NA%)	0/1 (0%)	1/1 (100%)	15/442 (3.4%)	18/466 (3.9%)

MLL Cohort

Characteristic	N	STAG2<br>386	RAD21<br>66	SMC1A<br>9	SMC3<br>13	multiple<br>5	Cohesin-WT<br>1,378	Overall<br>1,109
**Sex** *n/N (%)*	**440**							
Female		118/349 (34%)	33/65 (51%)	1/8 (12%)	7/13 (54%)	1/5 (20%)	0/0 (NA%)	160/440 (36%)
Male		231/349 (66%)	32/65 (49%)	7/8 (88%)	6/13 (46%)	4/5 (80%)	0/0 (NA%)	280/440 (64%)
Missing		37	1	1	0	0	1378	579
**Age at...** *Median (Range)*								
MDS diagnosis	**211**	73 (32, 90)	72 (45, 88)	54 (54, 54)	73 (66, 83)	80 (57, 90)	NA	73 (32, 90)
AML diagnosis	**769**	69 (27, 90)	63 (21, 88)	65 (21, 78)	60 (53, 73)	74 (73, 76)	68 (2, 93)	68 (2, 93)
**MDSWHO2022, n / N (%)**								
within subunit	**211**	**185 / 386 (48%)**	**14 / 66 (21%)**	**1 / 9 (11%)**	**8 / 13 (62%)**	**3 / 5 (60%)**	**838 / 1,378 (60%)**	**211 / 1,109 (19%)**
MDS-5q		1 / 185 (0.5%)	2 / 14 (14%)	0 / 1 (0%)	0 / 8 (0%)	0 / 3 (0%)	33 / 838 (3.9%)	36 / 1,049 (3.4%)
MDS-biTP53		0 / 185 (0%)	0 / 14 (0%)	0 / 1 (0%)	0 / 8 (0%)	0 / 3 (0%)	37 / 838 (4.4%)	37 / 1,049 (3.5%)
MDS-IB1		73 / 185 (39%)	4 / 14 (29%)	0 / 1 (0%)	2 / 8 (25%)	1 / 3 (33%)	157 / 838 (19%)	237 / 1,049 (23%)
MDS-IB2		76 / 185 (41%)	2 / 14 (14%)	1 / 1 (100%)	2 / 8 (25%)	1 / 3 (33%)	121 / 838 (14%)	203 / 1,049 (19%)
MDS-LB		27 / 185 (15%)	5 / 14 (36%)	0 / 1 (0%)	1 / 8 (12%)	1 / 3 (33%)	242 / 838 (29%)	276 / 1,049 (26%)
MDS-SF3B1		8 / 185 (4.3%)	1 / 14 (7.1%)	0 / 1 (0%)	3 / 8 (38%)	0 / 3 (0%)	248 / 838 (30%)	260 / 1,049 (25%)
**AML WHO2020, n / N (%)**								
within subunit	**769**	**164 / 386 (42%)**	**51 / 66 (77%)**	**7 / 9 (78%)**	**5 / 13 (38%)**	**2 / 5 (40%)**	**540 / 540 (100%)**	**769 / 1109 (69%)**
AML by differentiation		0 / 164 (0%)	12 / 51 (24%)	2 / 7 (29%)	0 / 5 (0%)	0 / 2 (0%)	33 / 540 (6.1%)	47 / 769 (6.1%)
AML with biallelic CEBPA		4 / 164 (2.4%)	1 / 51 (2.0%)	0 / 7 (0%)	0 / 5 (0%)	0 / 2 (0%)	41 / 540 (7.6%)	46 / 769 (6.0%)
AML with CBFB-MYH11		0 / 164 (0%)	0 / 51 (0%)	0 / 7 (0%)	0 / 5 (0%)	0 / 2 (0%)	41 / 540 (7.6%)	41 / 769 (5.3%)
AML with DEK-NUP214		0 / 164 (0%)	0 / 51 (0%)	0 / 7 (0%)	0 / 5 (0%)	0 / 2 (0%)	12 / 540 (2.2%)	12 / 769 (1.6%)
AML with GATA2, MECOM		1 / 164 (0.6%)	0 / 51 (0%)	0 / 7 (0%)	0 / 5 (0%)	0 / 2 (0%)	53 / 540 (9.8%)	54 / 769 (7.0%)
AML with KMT2A-MLLT3		0 / 164 (0%)	0 / 51 (0%)	0 / 7 (0%)	0 / 5 (0%)	0 / 2 (0%)	30 / 540 (5.6%)	30 / 769 (3.9%)
AML with NPM1		5 / 164 (3.0%)	22 / 51 (43%)	1 / 7 (14%)	3 / 5 (60%)	1 / 2 (50%)	116 / 540 (21%)	148 / 769 (19%)
AML with PML-RARA		0 / 164 (0%)	0 / 51 (0%)	0 / 7 (0%)	0 / 5 (0%)	0 / 2 (0%)	43 / 540 (8.0%)	43 / 769 (5.6%)
AML with RUNX1-RUNX1T1		0 / 164 (0%)	8 / 51 (16%)	1 / 7 (14%)	0 / 5 (0%)	0 / 2 (0%)	25 / 540 (4.6%)	34 / 769 (4.4%)
AML-MR		154 / 164 (94%)	8 / 51 (16%)	3 / 7 (43%)	2 / 5 (40%)	1 / 2 (50%)	146 / 540 (27%)	314 / 769 (41%)
**MDS/MPN** *n/N (%)*								
within subunit	**39**	**37 / 386 (10%)**	**1 / 66 (2%)**	**1 / 9 (11%)**	**0 / 13 (0%)**	**0 / 5 (0%)**	**0 / 540 (0%)**	**39 / 1109 (4%)**
aCML/BCR-ABL1 negative		9/37 (24%)	0/1 (0%)	0/1 (0%)	0/0 (NA%)	0/0 (NA%)	0/0 (NA%)	9/39 (23%)
CMML		14/37 (38%)	1/1 (100%)	0/1 (0%)	0/0 (NA%)	0/0 (NA%)	0/0 (NA%)	15/39 (38%)
MDS/MPN-u		14/37 (38%)	0/1 (0%)	1/1 (100%)	0/0 (NA%)	0/0 (NA%)	0/0 (NA%)	15/39 (38%)
